# Rrm2b deletion causes mitochondrial metabolic defects in renal tubules

**DOI:** 10.1038/s41598-019-49663-3

**Published:** 2019-09-13

**Authors:** Yi-Fan Chen, I-Hsuan Lin, Yu-Ru Guo, Wei-Jun Chiu, Mai-Szu Wu, Wei Jia, Yun Yen

**Affiliations:** 1 The Ph.D. Program for Translational Medicine, College of Medical Science and Technology, Taipei Medical University, 11031 Taipei, Taiwan; 2 TMU Research Center of Cancer Translational Medicine, Taipei Medical University, 11031 Taipei, Taiwan; 3 Ph.D. Program of Cancer Biology and Drug Discovery, College of Medical Science and Technology, Taipei Medical University, 11031 Taipei, Taiwan; 4 Department of Internal Medicine, School of Medicine, College of Medicine, Taipei Medical University, 11031 Taipei, Taiwan; 5 Division of Nephrology, Department of Internal Medicine, Taipei Medical University-Shuang Ho Hospital, 23561 New Taipei City, Taiwan; 6 Cancer Biology Program, University of Hawaii Cancer Center, Honolulu, HI 96813 USA

**Keywords:** Metabolomics, Genetics, Kidney

## Abstract

Renal diseases impose considerable health and economic burdens on health systems worldwide, and there is a lack of efficient methods for the prevention and treatment due to their complexity and heterogeneity. Kidneys are organs with a high demand for energy produced by mitochondria, in which Rrm2b has critical functions as reported. The Rrm2b kidney-specific knockout mice we generated exhibited age-dependent exacerbated features, including mitochondrial dysfunction and increased oxidative stress; additionally, resulted in severe disruption of mitochondria-related metabolism. Rrm2b is vital not only to supply dNTPs for DNA replication and repair, but also to maintain structural integrity and metabolic homeostasis in mitochondria. Thence, Rrm2b deletion might induce chronic kidney defects in mice. This model can facilitate exploration of novel mechanisms and targeted therapies in the kidney diseases and has important translational and clinical implications.

## Introduction

Ribonucleotide reductase (an α2β2 tetramer composed of two non-identical subunits) catalyzes the rate-limiting step in the *de novo* reduction of ribonucleoside diphosphates to deoxyribonucleotide triphosphates (dNTPs) during DNA synthesis and repair in the nucleus and mitochondria. Ribonucleotide reductase M2B (Rrm2b), also known as p53R2, is a subunit of the ribonucleotide reductase complex. Rrm2b is a p53-inducible gene that was first identified in cancer-derived human cells with a highly regulated p53 expression system^1–3^. Rrm2b plays critical roles in DNA repair, mitochondrial DNA (mtDNA) synthesis, oxidative stress resistance, cell cycle regulation and metastasis suppression^3–8^. Kimura and colleagues generated Rrm2b-deficient mice, which developed normally until weaning and then showed growth retardation and early mortality^9^. Severe renal failure, caused by activation of p53-dependent apoptosis, was found in Rrm2b-deficient mice. Thence, Rrm2b seems to have critical function in kidney, but its mechanism is unclear. Rrm2b can resist oxidative stress by scavenging reactive oxygen species (ROS) and is involved in the control of mitochondria homeostasis, including structural integrity and functional capacity^6,10–13^. In Rrm2b knockout mouse embryonic fibroblasts (MEFs), dNTP pools were severely attenuated under oxidative stress conditions. Rrm2b deficiency also causes severe mtDNA deletion in various tissues in mice^4^. Recent studies have unveiled that cooperation between RRM2B and ∆^1^-pyrroline-5-carboxylate reductases 1 and 2 (PYCRs) can directly or indirectly modulate antioxidative responses in human telomerase reverse transcriptase (hTERT)-immortalized normal human foreskin fibroblasts (HFFs-hTERT)^14^. Compromised DNA repair in the nucleus and mitochondria can result in profound DNA damage and affect mitochondrial homeostasis, which can subsequently increase oxidative stress and cellular damage. Rrm2b plays an important role in DNA repair and maintenance of mitochondrial functions. In this study, we generated a mouse model with conditional knockout of Rrm2b in the renal tubular epithelium to study the effects of Rrm2b deletion on mitochondria-related and renal functions. We propose that Rrm2b deletion damages mitochondrial integrity and impedes the mitochondrial functions, resulting in the disruption of the homeostasis of mitochondrial metabolisms.

## Materials and Methods

### Animals

An Rrm2b targeting vector containing two direct repeats of LoxP sites that flanked exon 3 to exon 5 of the Rrm2b gene was used as a set of homologous recombination arms to create an Rrm2b floxed allele (Rrm2b Flx/Flx; Rrm2b F/F). The targeted ES cell clones (C57BL/6 strain background) were screened by Southern blot analysis using designed probes. In Cdh16-Cre transgenic mice purchased from the Jackson Laboratory (JAX 012237)^15^, the Cdh-16 (also known as Ksp-cadherin) promoter was activated in the tubular epithelial cells of the kidney^16^. Ksp-cadherin was found to be expressed in the basolateral membrane of renal tubular epithelial cells and collecting duct cells, but not in glomeruli, blood vessels, or renal interstitial cells. After two generations of breeding with the Rrm2b F/F mice, kidney-specific Rrm2b KO mice were obtained and verified using regular PCR genotyping. The mice were bred and maintained in a specific pathogen-free facility. Euthanasia was performed using CO_2_ inhalation. The animal protocol was approved by the Institutional Animal Care and User Committee of National Defense Medical Center and all methods were performed in accordance with the relevant guidelines and regulations.

### RNA analysis

Total RNA was isolated from the kidney cortex using TRIzol Reagent (Life Technology). We performed real-time quantitative PCR using a TaqMan probe with TaqMan^®^ Fast Universal PCR Master Mix and a real-time PCR instrument (Roche Life Science; Thermo-Fisher Scientific). Amplification was executed in triplicates for each RNA sample and primer set.

### Protein and metabolite analysis

Tissue samples were homogenized in lysis buffer with complete protease inhibitor and phosphatase inhibitor cocktails (Roche) and then denatured in SDS sample buffer in a boiling water bath. The total extracted proteins were separated on an SDS–polyacrylamide gel (Bio-Rad) and transferred to a Hybond N+ membrane (GE Healthcare). The membranes were blocked with 5% (w/v) nonfat dry milk, incubated with primary antibodies against Rrm2b (1:2000, GTX109620, GeneTex) and Hsp70 (1:5000, GTX111088, GeneTex) and washed, and the proteins were detected using a Visualizer Kit (Millipore). Free glycerol in the serum and urine were measured calorimetrically using commercially available kits (Randox Lab Ltd.).

### Histopathology

Tissues were fixed in formalin buffered with phosphate and embedded in paraffin. Tissue sections (4 µm) were subjected to hematoxylin-eosin (H&E), immunohistochemistry (IHC) and special staining following standard procedures^17^. The IHC staining was performed by incubating sections with primary antibodies, including antibodies targeting Rrm2b (1:200, PAB12860, Abnova), 4-Hydroxynonenal (4-HNE) (1:100, ab46545, abcam), and ki67 (1:50, 550609, BD Pharmingen), for 18–24 h at 4 °C, and detection was performed using biotinylated secondary antibodies and an LSAB Kit (DakoCytomation). The specific staining for kidney sections was performed using a Gomori trichrome staining kit (Polysciences, Inc.) and Picosirius red staining kit (Atom Scientific).

### Transmission electron microscopy (TEM)

Mouse tissues were fixed in cacodylate buffer with glutaraldehyde (1.5%) and paraformaldehyde (1.5%), post-fixed in 1% OsO4 and rinsed in cacodylate buffer. Following dehydration, tissues were embedded in Epon and sectioned for TEM. The tissue ultrastructure was observed using a transmission electron microscope (TEM HT7700, HITACHI, Tokyo, Japan).

### Mitochondria analysis

Oxygen consumption rate (OCR) of isolated mitochondria from tissues (kidney cortex) were measured using a Seahorse XFe Extracellular Flux Analyzer (Seahorse bioscience), and the results were normalized according to the protein contents of each sample. Mitochondria isolation from tissues was performed as previously described^18^. Mitochondrial genomic DNA was co-purified with nuclear genomic DNA, and quantitative real-time PCR was performed for detection of mt-Co2 (Cytochrome c oxidase subunit 2; mitochondrial DNA-encoded) and 18S rRNA (18S ribosomal RNA; nuclear DNA-encoded). The relative mtDNA copy number was calculated via normalization to 18S rRNA gene expression (mtDNA/nDNA)^19,20^.

### Extraction of metabolites

Sample preparation and metabolite quantification were performed according to methods described previously^21–24^. Briefly, the frozen tissue (50 ~ 100 mg) was homogenized on ice in a 500-µL mixture of chloroform, methanol and water (1:2:1, v/v/v) and centrifuged at 13,000 rpm for 10 min at 4 °C, and 150 µL of the supernatant was subsequently transferred to a sample vial. The remaining pellet was rehomogenized in 500 µL of methanol, followed by a second centrifugation. Another 150 µL aliquot of supernatant was added to the same sample vial, and then, the sample was dried. The dried extract was then derivatized with 80 µL of methoxyamine (15 mg/mL in pyridine) at 30 °C for 90 min. A 10-µL aliquot of retention index compound mixture (mixture of C10-C40, 50 µg/mL) was added to the derivatized sample. The second step of the derivatization was performed by adding 80 µL of N-O-bis-(trimethylsilyl)-trifluoroacetamide (BSTFA) (1% trimethylchlorosilane) and incubating the sample at 70 °C for 120 min. The samples were subjected to gas chromatography-time of flight mass spectrometry (GC-TOF-MS) analysis directly after derivatization.

### GC-TOF-MS standards

Mammalian metabolite standards were obtained from Sigma-Aldrich (St. Louis, MO), Santa Cruz Biotech. (Dallas, TX) or Avanti polar lipids (Alabaster, AL). The standards (approximately 1,000) were prepared in appropriate solutions and analyzed by GC-TOFMS to establish an in-house metabolite database.

### GC-TOFMS analysis

The separation analysis was performed using an Agilent 7890N gas chromatograph in splitless mode connected to a time-of-flight mass spectrometer (LECO Corp., St. Joseph, MI). Separation was achieved using an Rxi-5ms capillary column (Crossbond® 5% diphenyl/95% dimethyl polysiloxane; Restek), with helium as the carrier gas at a constant flow rate of 1.0 mL/min. The GC oven temperature program was as follows: 2 min at 80 °C, raised by 10 °C/min to 140 °C, by 4 °C/min to 180 °C, by 10 °C/min to 240 °C and by 25 °C/min to 290 °C, followed by hold for 4.5 min at 290 °C. The temperatures of the injection, transfer interface, and ion source were set to 270, 270, and 220 °C, respectively. Mass spectra were obtained with electron impact ionization (70 eV) at full scan mode (m/z 30–600) and an acquisition rate of 25 spectra/s.

### Analysis of mass spectra

Data acquisition was performed using ChromaTOF software (v4.22, LECO Corp., St. Joseph, MI). MATLAB (v7.0, Mathworks, Natick, MA) software was used to perform data pretreatment procedures, such as baseline correction, de-noising, smoothing, alignment, time-window splitting, and multivariate curve resolution^25^. Internal standards and any known artificial peaks, such as peaks caused by noise, column bleed and the BSTFA derivatization procedure, were removed from the data set. Metabolites were annotated through comparison with the reference standards in our in-house library. Commercially available mass spectral databases, such as the NIST library 2010 and the LECO/Fiehn Metabolomics Library, were also used for additional compound annotation (with a similarity threshed of 70%). The mass spectral data were subjected to log2 transformation and normalized by autoscaling.

### Statistical analysis

All results are presented as the mean ± SD of at least three independent samples. Comparisons between two groups were calculated using Student’s t test. Statistical differences between two groups were analyzed, and the differences were considered significant when p < 0.05.

## Results

### Generation and characteristics of the Rrm2b kidney-specific knockout mouse model

**Figure 1:**
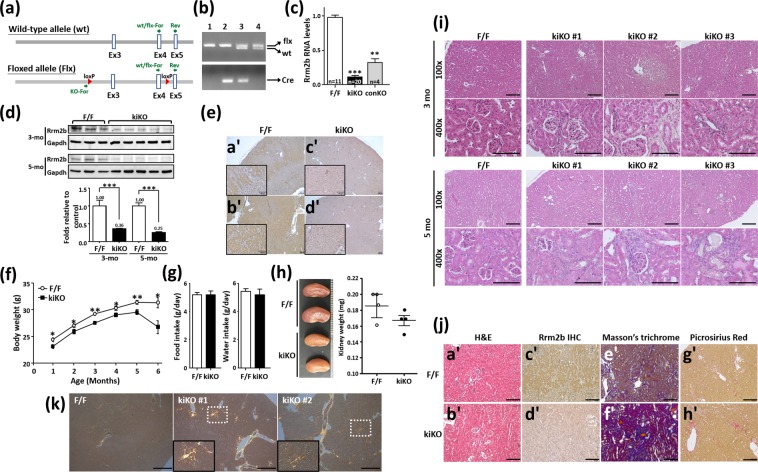
Physical characterization of Rrm2b kidney-specific knockout mice. (**a)** The location of two loxP sites in cis and the primers for genotyping to verify Rrm2b floxed, wild-type and knockout allele. **(b)** Genotyping of floxed and wild-type alleles using PCR. Genotype of sample 1 was wild-type (+/+); genotype of sample 2 was Cdh16-Cre/+; genotype of sample 3 was Rrm2b kiKO (Rrm2b flx/flx;Chd16-Cre/+); genotype of sample 4 was Rrm2b flx/flx (F/F). **(c)** Quantitative real-time PCR analysis of Rrm2b in kidney from Rrm2b kiKO and control (F/F) mice. conKO, Rrm2b conventional knockout mice. **(d)** Western blot analysis of Rrm2b in kidney from Rrm2b kiKO and control (F/F) mice. Quantification of western blot analysis by Image J software. **(e)** IHC staining of Rrm2b in kidney from Rrm2b kiKO and control (F/F) mice. Mouse age in (**c–e**), 2 months old. Scale bar, 100μm. **(f)** Examination of body weight. The data were collected from 1- to 6-month-old male mice (n = 8–30 per group). **(g)** Food and water intake were monitored using metabolic cages. A total of 10 mice from each group were used for experimentation. **(h)** Gross view and quantified mass of kidneys from Rrm2b kiKO and F/F (control) mice. **(i)** H&E staining of kidneys from F/F (control) and Rrm2b kiKO mice. **(j)** Collagen accumulation was observed with Rrm2b deletion: (a’,b’) H&E staining, (c’,d’) Rrm2b IHC staining, (e’,f’) Masson’s trichrome staining, (g’,h’) Picrosirius Red staining. **(k)** Polarization contrast of the photos stained with picrosirius red. Enlarged view indicated the collagen-accumulated positions. Mouse age, 5–6 months old. Scale bar, 100 μm. The results are presented as the mean ± SD. *p < 0.05; **p < 0.005; ***p < 0.001.

The tubular cells of the renal cortex are rich in mitochondria, for the purpose of energy production and maintenance of tubular functions. To study the involvement of Rrm2b in renal functions, we generated renal tubular epithelial cell-specific Rrm2b knockout mice (Rrm2b^flx/flx^; Cdh16-Cre/+, abbreviated to Rrm2b kiKO) (Fig. 1a,b). Western blot analysis, quantitative real-time PCR and IHC staining demonstrated that Rrm2b was abundantly expressed in normal renal tubular cells and was mostly absent in Rrm2b kiKO mice (Fig. 1c–e). Rrm2b kiKO male mice exhibited significant growth retardation from ages 1 to 6 months (Fig. 1f), without a decrease in the daily intake of food and water (Fig. 1g). Serum biochemical indices were monitored and showed no obvious differences between Rrm2b kiKO mice and control Rrm2b^flx/flx^ (F/F) mice at 3 and 6 months of age (Supplemental Fig. S1a–f). Urinary biochemical data also showed no apparent changes, except for a decrease in the pH value (Rrm2b kiKO, pH = 5.81 *vs*. Rrm2b F/F, pH = 6.25, p = 0.026) and a slight increase in the ketone concentration in 6-month-old kiKO mice compared with that in Rrm2b F/F mice. (Table 1). However, after fasting, Rrm2b deleted mice showed slightly lower serum insulin levels but not significant. Additionally, there were no significant differences in the blood glucose and fasting blood glucose levels between Rrm2b deleted model and control mice (Supplemental Fig. S1g–i). Pale and smaller kidneys were found in Rrm2b kiKO mice (Fig. 1h). Tubule dilation and degeneration and damage-induced inflammation were observed in the renal cortex of Rrm2b kiKO mice (Figs 1i, S2a,b). A clear sign of inflammation was also observed in female mice with Rrm2b deletion (Supplemental Fig. S2c). A marginal accumulation of collagen was also observed in the kidneys of Rrm2b kiKO mice (Fig. 1j). Additionally, sirius red sections were viewed with polarization contrast illumination suggesting the collagen accretion was dominant in Rrm2b kiKO kidney (Fig. 1k). The defects observed in the kidneys are most likely the results of the Rrm2b deficiency causing an age-dependent deterioration in kidney function. Rrm2b has also been shown to be involved in decreasing ROS activity to reduce oxidative stress. Our data showed that *Rrm2b* deletion resulted in enhanced expression of ROS-associated genes, such as transformation related protein 53 (*Trp53*) and apurinic/apyrimidinic endonuclease 1 (*Apex1/Ref1*) (Fig. 2a), and reduced the expression of antioxidants, such as catalase (*Cat*) and superoxide dismutase 1 (*Sod1*) (Fig. 2b), in Rrm2b kiKO kidneys relative to control Rrm2b F/F kidneys. The oxidative stress marker 4-HNE, a by-product of lipid peroxidation, was found to have significantly increased expression levels in Rrm2b knockout renal tissue (Fig. 2c). Interestingly, *Pycr1* expression was dramatically increased in Rrm2b kiKO mice, and a slight increase in nuclear factor erythroid derived 2 like 2 (*Nef2l2* or *Nrf2*) was also observed (Fig. 2d). We also found induced expression of genes associated with cell proliferation, such as cadherin 1 (*Cdh1*) and proliferating cell nuclear antigen (*Pcna*) (Fig. 2e), in Rrm2b kiKO mice, and increased cell proliferation was confirmed with IHC staining for ki67 (Fig. 2f). Therefore, Rrm2b deficiency not only promotes ROS accumulation but also weakens cellular antioxidant capacity.

**Table 1:** Biochemical indices in urine of Rrm2b kiKO mice.

Age (months)	Genotype	Glucose	Bilirubin	Urobilinogen (mg/dl)**	pH	S.G. (Specific Gravity)	Blood	Ketones (mg/dl)#	Nitrite	Leukocytes	Color
3	F/F	−	−	++ (4)	6	>1.030	−	+/−	−	−	Yellow
		−	−	++ (4)	7	1.025	−	+/−	−	−	Yellow
		−	−	++ (4)	6.5	>1.030	−	+ (10)	−	−	Yellow
		−	−	++ (4)	6.5	>1.030	−	+/−	−	−	Yellow
		−	−	++ (4)	6.5	>1.030	−	+ (10)	−	−	Yellow
		−	−	++ (4)	6.5	>1.030	−	+ (10)	−	75 Leu/UI	Yellow
	kiKO	−	−	+ (3)	6	>1.030	−	+/−	−	−	Yellow
		−	−	+ (3)	6	>1.030	−	+/−	−	−	Yellow
		−	−	++ (4)	6	>1.030	−	+ (10)	−	−	Yellow
		−	−	++ (4)	6	>1.030	−	+ (10)	−	−	Yellow
		−	−	+ (2)	6	1.025	−	+/−	−	−	Yellow
		−	−	++ (4)	6	>1.030	−	+/−	−	−	Yellow
6	F/F	−	−	+ (3)	6.5	>1.030	−	+ (10)	−	−	Yellow
		−	−	++ (4)	6	>1.030	−	+ (10)	−	−	Yellow
		−	−	+ (3)	6.5	>1.030	−	+ (10)	−	−	Yellow
		−	−	++ (6)	6.5	>1.030	−	+ (10)	−	−	Yellow
		−	−	+ (3)	6	>1.030	−	+/−	−	−	Yellow
		−	−	++ (4)	6	>1.030	−	+ (10)	−	−	Yellow
	kiKO	−	−	+ (3)	5.5	>1.030	−	+ (10)	−	−	Yellow
		−	−	++ (6)	5.5	>1.030	−	+ (20)	−	−	Yellow
		−	−	++ (4)	6	>1.030	−	+ (10)	−	−	Yellow
		−	−	++ (4)	6	>1.030	−	+ (10)	−	−	Yellow
		−	−	++ (4)	5.5	>1.030	−	+ (20)	−	−	Yellow
		−	−	+ (3)	5.5	>1.030	−	+ (10)	−	−	Yellow
		−	−	+ (3)	6	>1.030	−	+/−	−	−	Yellow
		−	−	++ (6)	6.5	>1.030	−	+ (10)	−	25 Leu/ul	Yellow

**Urobilinogen: 1+, 2–3; 2+, 4–6; 3+, 8–12.

#Ketones:±, <10; 1+, 10–20; 2+, 40–60.

**Figure 2:**
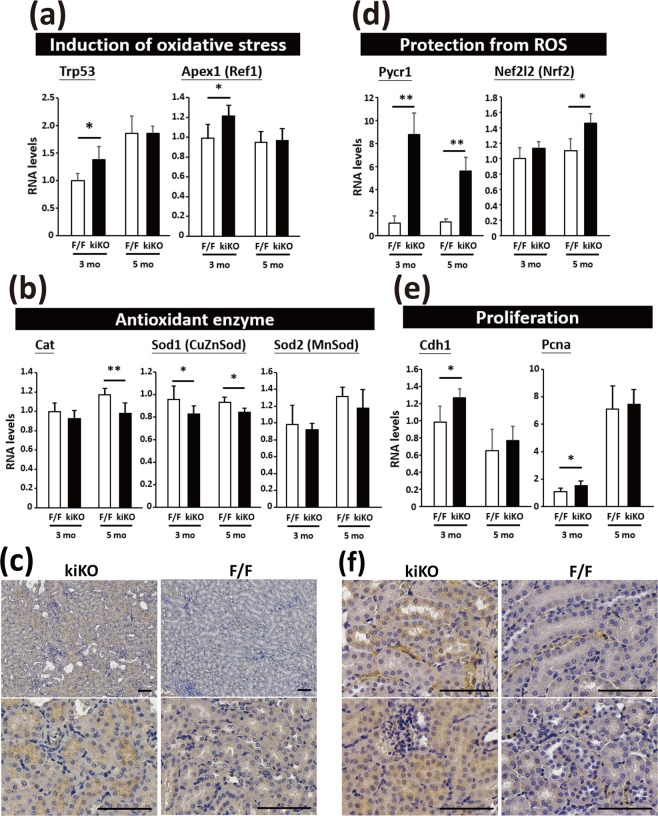
Elevated ROS and cell proliferation in kidney of Rrm2b kidney-specific knockout mice. (**a)** The mRNA expression levels of genes related to induce oxidative stress or associated genes. *Trp53*, transformation related protein 53; *Apex1* (*Ref1*), apurinic/apyrimidinic endonuclease 1. **(b)** The mRNA expression levels of enzymes related to antioxidant defense. *Cat*, catalase; *Sod1* (*CuZnSod*), superoxide dismutase 1, soluble; *Sod2* (*MnSod*), superoxide dismutase 2, mitochondrial. **(c)** IHC staining of 4-HNE, an oxidative stress marker, indicated the tissue part with oxidative damage. **(d)** The mRNA expression levels of *Pycr1*, pyrroline-5-carboxylate reductase 1 and *Nef2l2* (*Nrf2*), nuclear factor, erythroid derived 2, like 2. **(e)** The mRNA expression levels of genes related to cell proliferation. *Cdh1*, cadherin 1; *Pcna*, proliferating cell nuclear antigen. **(f)** IHC staining of ki67 in Rrm2b kiKO and control mice at 3 months old. Scale bar, 100μm. The results are presented as the mean ± SD. *p < 0.05; **p < 0.005.

### Rrm2b deletion induces defects in mitochondrial functions

**Figure 3:**
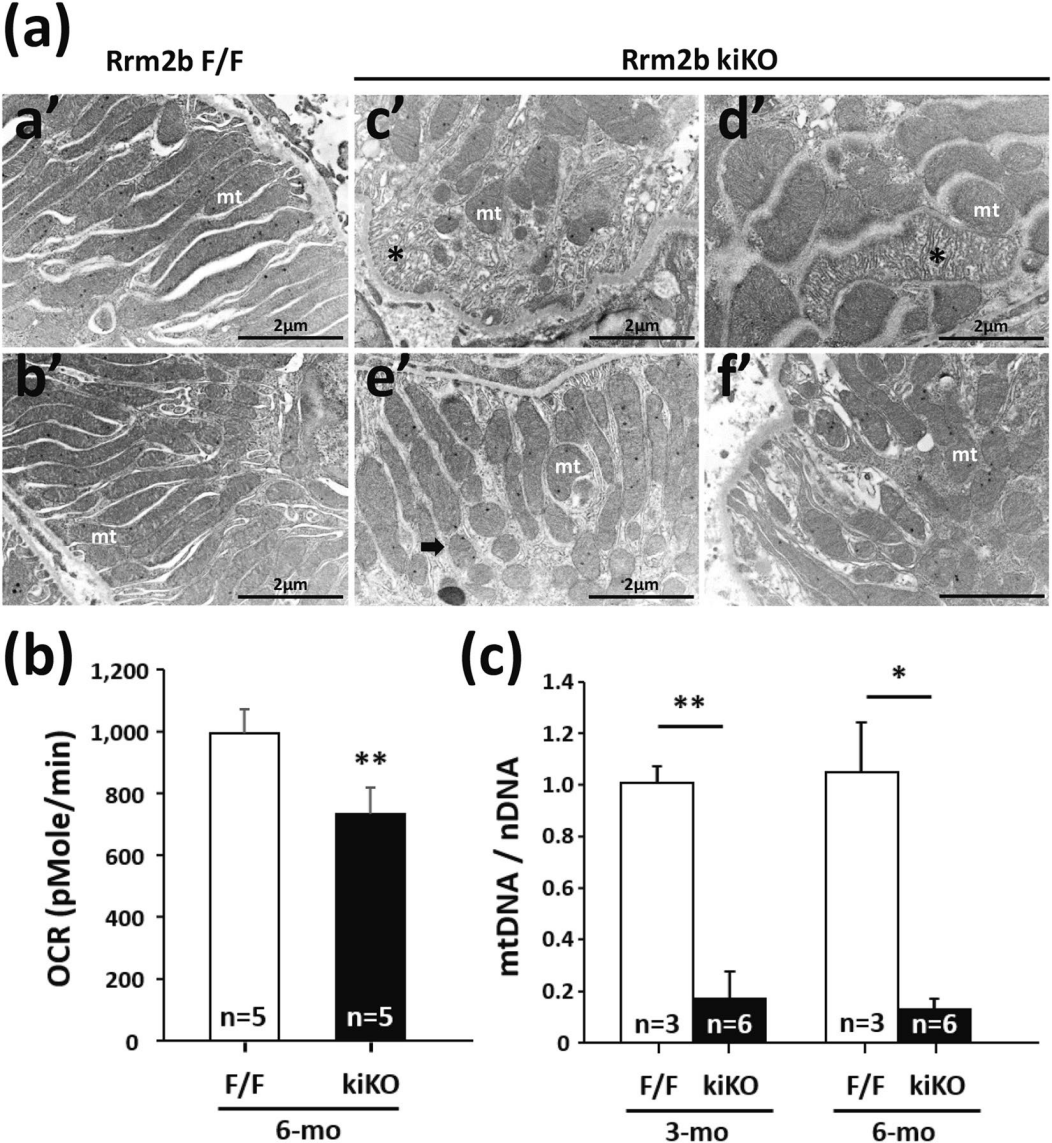
Mitochondria defects in Rrm2b kidney-specific knockout mice. (**a)** Ultrastructure of kidney cortex. (a’,b’) Numerous elongated mitochondria (mt) were located in proximity to the plasma membrane of cell in wild-type proximal tubule. (c’–f’) Fragmented (arrow head) and degenerated (asterisk) mitochondria were found in the proximal tubules of Rrm2b deleted kidney. Mouse age, 3 months old. **(b)** Analysis of the efficiency of oxygen consumption in kidney cortex from 6-month-old mice. **(c)** Mitochondrial DNA (*mt-Co2*; mitochondria-encoded gene; mtDNA) was measured relative to *18S rRNA* (nucleus-encoded gene; nDNA) as the control. The results are presented as the mean ± SD. *p < 0.05; **p < 0.005.

The mitochondria in the proximal renal tubular cells of the wild-type kidneys showed typical basal striping characteristics (Fig. 3a). In Rrm2b kiKO mice, fragmented and degenerated mitochondria were found in proximal renal tubular cells (Fig. 3a), although the genes related to mitochondrial fusion and fission showed no obvious expression level differences between Rrm2b kiKO and F/F mice (Supplemental Fig. S3a–c). These data indicate that Rrm2b deletion caused mitochondrial breakdown that was not due to the effects of mitochondrial fusion and fission. Maintenance of renal function is highly dependent on mitochondrial energy production. We found that Rrm2b deletion resulted in a decrease in oxygen consumption (Fig. 3b), which can greatly impede energy production in mitochondria. Additionally, the number of mitochondria was reduced significantly in Rrm2b knockout kidney tissue (Fig. 3c). These data provide evidence supporting the notion that Rrm2b plays a vital role in mitochondrial homeostasis.

### Defects in mitochondria-involved metabolism in Rrm2b kiKO mice

To systematically understand the effects of Rrm2b-deletion in the kidney, we performed metabolic profiling analysis of the kidneys of Rrm2b kiKO and F/F mice. GC-TOF-MS analysis identified a total of 126 metabolites; of these, 13 metabolites exhibited lower concentrations and 14 exhibited higher concentrations in Rrm2b kiKO mice than in control mice (Table 2; Supplemental Table S1). In the Rrm2b kiKO kidneys, the level of glucose 6-phosphate, which is an important intermediate of the glycolysis and pentose phosphate pathways, was increased (2.6-fold) compared with that in control kidneys, and the levels of glycerol 3-phosphate and glycerol concentrations were also increased (1.27- and 1.88-fold, respectively) compared with those in control kidneys, possibly through glyceroneogenesis. When the metabolites of methionine and cysteine metabolism were examined, homocysteine, homoserine and serine concentrations were found to be lower (0.84-, 0.52- and 0.77-fold, respectively) and dimethylglycine was higher (1.27-fold) in Rrm2b kiKO mice than in control mice. Two key substrates of the citrate cycle (TCA cycle), oxoglutaric acid and succinic acid, were also found at lower concentrations (0.74- and 0.78-fold, respectively) in Rrm2b deleted kidneys relative to control (F/F) kidneys. Lastly, the caffeine metabolite 7-methylxanthine was found at higher concentrations (1.72-fold) in Rrm2b kiKO mice than in control mice (Supplemental Table S1).

**Table 2:** Metabolites identified from mouse kidney with GC-TOF-MS.

Human Metabolome Database (HMDB) Class	Total Counts	Rrm2b kiKO vs. Control		
		Down^a^	Up^b^	No Change
Organoheterocyclic compounds	8	0	0	8
Benzenoids	2	0	0	2
Homogeneous non-metal compounds	1	0	0	1
Lipids and lipid-like molecules	23	1	1	21
Nucleosides, nucleotides, and analogues	6	1	2	3
Organic acids and derivatives	51	6	5	40
Organic nitrogen compounds	2	0	0	2
Organic oxygen compounds	24	4	5	15
Organoheterocyclic compounds	8	1	1	6
NA	1	0	0	1
Total Metabolites	126	13	14	99

^a^Rrm2b kiKO has lower concentration: log2 fold change < −0.25.

^b^rm2b kiKO has higher concentration: log2 fold change > 0.25.

### Altered expression of key metabolic genes in Rrm2b kiKO mice

**Figure 4:**
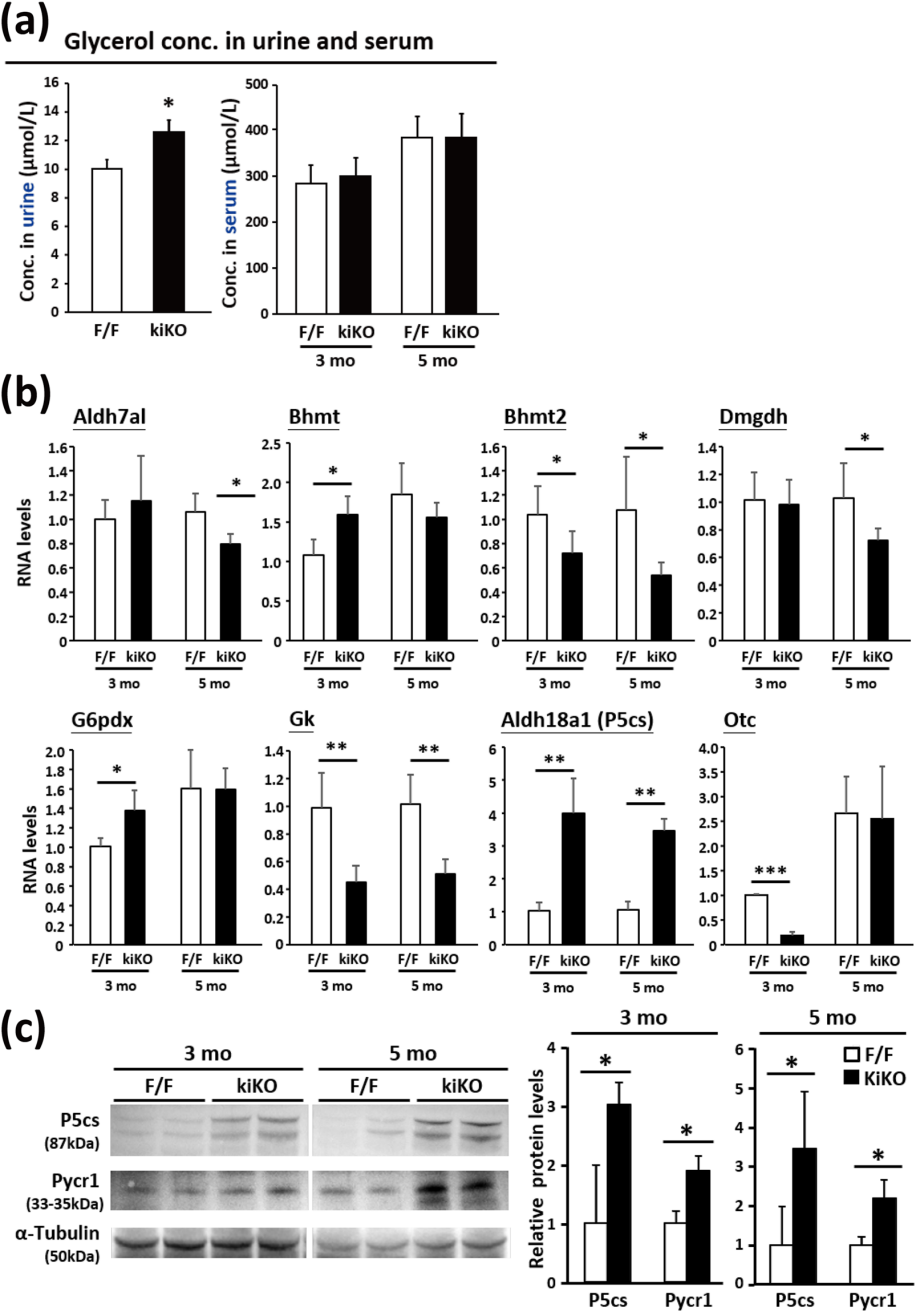
The effects of Rrm2b deficiency on mitochondria-related metabolism. **(a)** The glycerol concentration in urine and serum was measured using a glycerol assay kit. **(b)** Quantification of the expression levels of key genes involved in mitochondria-related metabolism. **(c)** Western blot analysis of P5cs and Pycr1 in Rrm2b kiKO and WT mice at 3 and 5 months of age. α-Tubulin expression levels as internal control for quantifying of P5cs and Pycr1 expression. *Aldh7a1*, aldehyde dehydrogenase family 7, member A1; *Aldh18a1*, aldehyde dehydrogenase 18 family, member A1 (also known as *P5cs*, pyrroline 5 carboxylate synthase); *Ass1*, argininosuccinate synthase; *Bhmt*, betaine-homocysteine methyltransferase; *Bhmt2*, betaine-homocysteine methyltransferase 2; *Cbs*, cystathionine beta-synthase; *Cth*, cystathionine gamma-lyase; *Dld*, dihydrolipoamide dehydrogenase; *Dmgdh*, dimethylglycine dehydrogenase precursor; *G6pdx*, glucose-6-phosphate dehydrogenase X-linked; *Gk*, glycerol kinase; *Gss*, glutathione synthase; *Idh*, isocitrate dehydrogenase; *Mdh*, malate dehydrogenase; *Mtr*, 5-methyltetrahydrofolate-homocysteine methyltransferase; *Otc*, ornithine transcarbamylase; *Sord*, sorbitol dehydrogenase. All the results are presented as the mean ± SD. *p < 0.05; **p < 0.005; ***p < 0.001.

Rrm2b deficiency led to changes in mitochondrial metabolite concentrations; therefore, we wanted to know whether the genes involved in these enzymatic activities were also affected in Rrm2b kiKO mice. We searched the public database MGI-Mouse Gene Expression Database (GXD) and identified 342 genes that are annotated as expressed in the renal cortex, as confirmed by RNA *in situ*, *in situ* reporter (knock-in), IHC, western blot, RT-PCR and/or northern blot analysis. The prediction scores of MitoProt, TargetP, MitoFates and MitoMiner were used as evidence of mitochondrial localization. Of the 342 genes, 67 encoded candidate mitochondrial proteins (Supplemental Table S2). We used the KEGG Pathway Mapping tool to illustrate the top 15 metabolic pathways that included the highest number of candidate mitochondrial proteins and differential abundant metabolites (Supplemental Table S3). Using mouse metabolic pathways (map mmu01100) as an overview, we observed a few pathways that had both the enzymes and the metabolic compounds highlighted, and some were in close proximity to each other in the pathway maps (Supplemental Fig. S4). Some essential metabolites and related enzymes showed significant changes in the Rrm2b deleted kidney. These metabolites and enzymes were involved in vital biological pathways, such as glycerolipid metabolism; glycine, serine and threonine metabolism; glycolysis; oxidative phosphorylation; the pentose phosphate pathway; and other amino acid metabolism pathways. We performed ELISA assays and confirmed that the glycerol concentration was elevated in the urine but not in the serum of Rrm2b kiKO mice compared with control mice (Fig. 4a). In Fig. 4b, we showed that the expression levels of aldehyde dehydrogenase family 7, member A1 (*Aldh7a1*), betaine-homocysteine methyltransferase 2 (*Bhmt2*), dimethylglycine dehydrogenase precursor (*Dmgdh*) and glycerol kinase (*Gk*) responsible for glycolysis, glycerolipid and amino acid metabolism were repressed. Interestingly, an increase in *Bhmt* expression caused the high levels of dimethylglycine (*DMG*) that were observed under Rrm2b deficiency conditions (Fig. 4b, Supplemental Table S1). In addition, some enzymes downstream of the abovementioned pathways, such as glucose-6-phosphate dehydrogenase X-linked (*G6pdx*); aldehyde dehydrogenase 18 family, member A1 (*Aldh18a1*, also known as *P5cs*, pyrroline 5 carboxylate synthase/Pyrroline 5 carboxylate dehydrogenase); and ornithine transcarbamylase (*Otc*), were elevated (Fig. 4b). There were also enzymes that showed no obvious expression differences between Rrm2b kiKO and F/F mice (Supplemental Fig. S5). Finally, the protein expression levels of P5cs and Pycr1 were similar to mRNA levels (Fig. 4c). All of these results indicate that Rrm2b deletion affects the mitochondrial metabolic activities the kidney.

## Discussion

**Figure 5:**
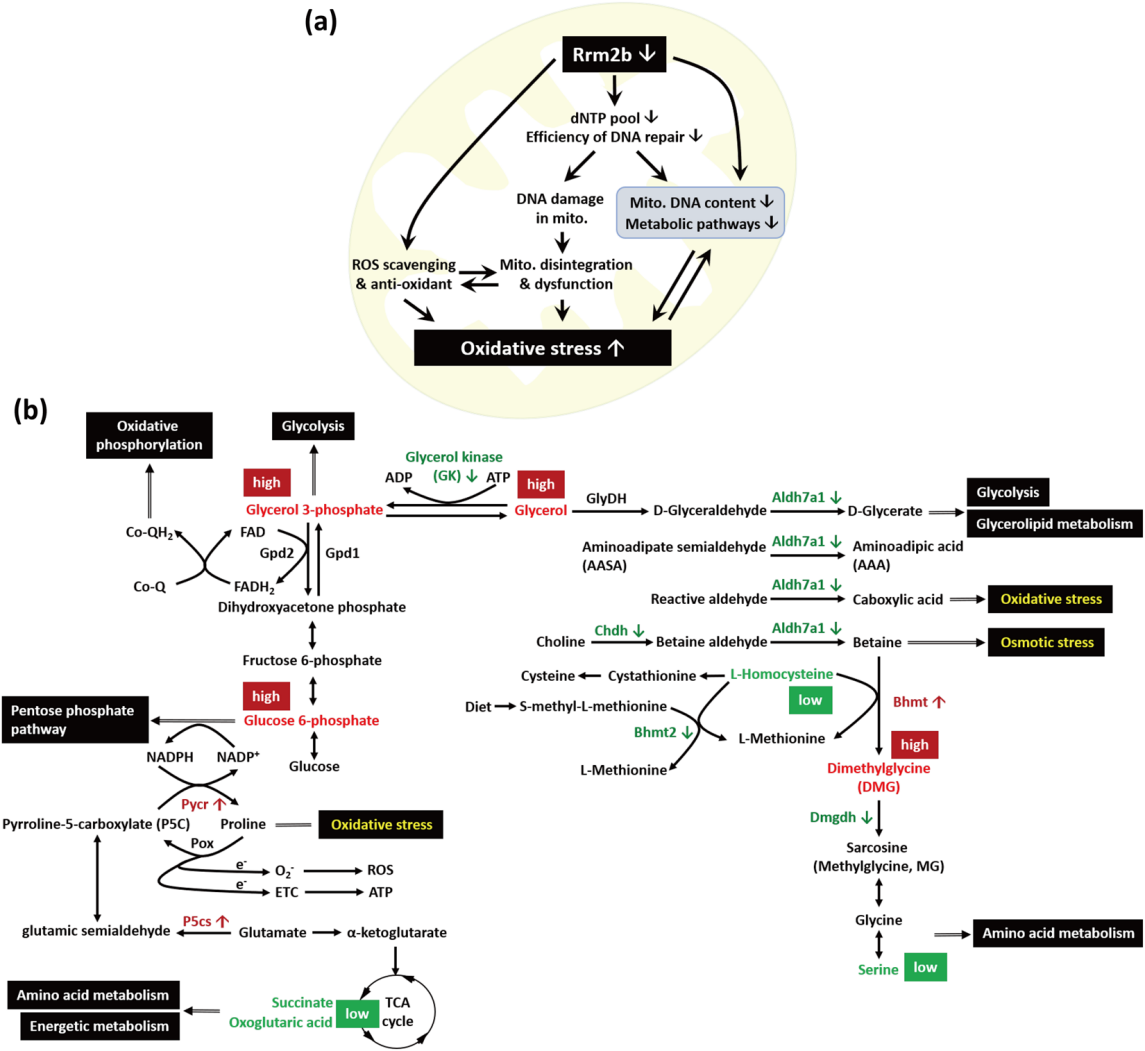
Schematic of mechanistic effects of Rrm2b deficiency in kidney. (**a)** The critical effects of Rrm2b deletion, based on previous reports and this study. **(b)** Schematic of pathways for Rrm2b-related metabolism under Rrm2b deficient condition. Red indicated the metabolites or enzymes that have higher expression, and green indicated the metabolites or enzymes that have lower expression in Rrm2b kiKO mice. Dimethylglycine dehydrogenase (Dmgdh; Me2GlyDH, matrix); Betaine homocysteine methltransferase (Bhmt, cytosol); Betaine homocysteine methltransferase 2 (Bhmt2, cytosol); NAD+-linked betaine aldehyde dehydrogenase (Aldh, matrix); Choline dehydrogenase (CHDH, inner cell membrane); Glycerol-3-phosphate dehydrogenase 1 (Gpd1, soluble, cytosol); Glycerol-3-phosphate dehydrogenase 2 (Gpd2, matrix); Glycerol dehydrogenase (Glydh).

Rrm2b plays pivotal roles in nuclear genomic DNA repair and replication and repair of mitochondrial genomic DNA^26^. Additionally, Rrm2b has the capacity to scavenge ROS to protect against oxidative stress^10^. Rrm2b-deficient human and mouse models exhibit detectable decreases in the dNTP pool and DNA repair efficiency, resulting in severe damage to nuclear and mitochondrial DNA^4,9^. In addition, loss of Rrm2b led to declines in ROS scavenging and antioxidant capacity, damaged mitochondrial structures and exacerbated impairment of mitochondrial functions. All of these effects accelerated cellular oxidative stress in organisms (Fig. 5a).

In this study, we generated a renal tubular epithelial cell-specific Rrm2b knockout model and showed that Rrm2b deletion affected mitochondrial DNA integrity and metabolism, causing elevated oxidative damage. By combining metabolomics and experimental results, we illustrated the pathways influenced by Rrm2b deletion (Fig. 5b). Gk is a mitochondrial-bound enzyme that is abundantly expressed in the kidney and liver and catalyzes the formation of glycerol 3-phosphate from ATP and glycerol^27^. Our data indicated that reduced energy production resulting from defective mitochondrial respiration drives an elevation in the levels of glycerol 3-phosphate and glucose 6-phosphate, which trigger energy-producing pathways, such as oxidative phosphorylation and glycolysis. In addition, the pentose phosphate pathway can also be activated to produce more nucleotides for DNA repair. Aldh7a1 (also known as antiquitin) is found in the cytosol, nucleus, and mitochondria and is highly expressed in the kidney, liver and brain. Biochemically, Aldh7a1 catalyzes the conversion of D-glyceraldehyde to D-glycerate, which can enter the glycolysis pathway. Aldh7a1 can also metabolize betaine aldehyde to betaine to counteract hyperosmotic stress^28,29^. Additionally, Aldh7a1 can attenuate osmotic stress-induced apoptosis. However, Rrm2b deletion led to reduced Aldh7a1 expression and induced extreme oxidative stress and osmotic stress. Bhmt and Dmgdh are expressed in the proximal tubules of the renal cortex^30,31^. Aberrant expression of these two proteins under Rrm2b deficiency conditions caused DMG accumulation and decreases in L-homocysteine (Hcy) and serine levels, which consequently disrupted amino acid metabolism (Fig. 5b).

Increased Pycr and P5cs expression levels facilitate proline generation and indirectly enhance ROS generation while reducing TCA cycle flux^32^. Expression of both Pycr1 and P5cs were markedly increased by 8- and 4-fold respectively in Rrm2b kiKO mice compared with control (Rrm2b F/F), while the proline oxidase (Pox) that converts proline to P5C showed no obvious difference in expression. The up-regulation of proline biosynthesis genes can trigger the conversion of glutamine towards proline and oxidizing NAD(P)H to NAD(P) + molecules. The activation of Pycr1 and P5cs resulted from Rrm2b deletion maybe a compensatory mechanism that cells used to counteract the increasing production of ROS.

### Phenotypes and related mechanisms

Based on our study, Rrm2b deletion in renal tubular cells caused several pathological defects in mice. Additionally, these defects were exacerbated with age, and some defects were similar to those observed in human disorders, as previously reported.

#### Changes in urine pH value

The proximal tubules regulate acid-base balance by secreting ammonia and reabsorbing filtrated bicarbonate. Urine biochemical analysis showed lower pH values in 6-month-old Rrm2b kiKO mice but not in younger mice; accordingly, we hypothesize that Rrm2b deletion may induce renal tubular acidemia type 2, also known as proximal renal tubular acidemia^33,34^. Human patients with this disease have a bicarbonate reabsorption defect in the kidney and intestine. Rrm2b deficiency in mice caused metabolic defects, functional loss of renal tubules, and maybe impaired bicarbonate reabsorption from urine in proximal tubular cells.

#### Mitochondrial defects mediate oxidative damage

The kidney is highly susceptible to ROS-induced damage. In addition, renal proximal tubular cells have abundant mitochondria and rely primarily on oxidative phosphorylation to obtain energy; thus, they are very sensitive to ROS-induced damage, such as mutation, apoptosis and mitochondrial-DNA-associated functional defects^35^. Although the effects of Rrm2b deficiency in the loop of Henle and collecting tubes are mostly unknown, oxidative damage mediated by mitochondria may lead to pathological changes in the loop of Henle and collecting tubes. Past studies have demonstrated that enhanced Rrm2b expression can reduce oxidative stress in both normal and oxidative-damage conditions^6,12^. Our data showed that Rrm2b deletion in renal tubular cells caused an increase in oxidative stress and a decrease in antioxidant capacity, which led to both morphological and functional mitochondrial defects. Additionally, exposure to oxidative stress can trigger increased expression of Pycr1^36,37^, and indeed, we found enhanced expression of Pycr1 and Nrf2, another oxidative stress-induced gene, in our Rrm2b kiKO model. This suggests that Rrm2b deletion caused oxidative damage in the kidney. One of the critical functions of Pycr1 is to maintain mitochondrial function and integrity under oxidative damage conditions^14,37^. However, the compensatory effects of Pycr1 and other stress-induced genes were not sufficient to counteract and protect mitochondria against oxidative damage under Rrm2b deletion conditions. Furthermore, impaired antioxidant mechanisms and reduced ROS clearance can lead to oxidative damage and mitochondrial breakdown.

#### Impaired mitochondrial metabolism

Unbalanced metabolism usually induces an overload of metabolites, accompanied by accumulation of cellular damage and overall cellular stress. Aldh7a1 protects cells against osmotic stress and thus attenuates osmotic stress-induced oxidative stress by metabolizing toxic aldehydes to produce NAD(P)H, which helps restore the cellular redox balance^29^. Under Rrm2b-deficient conditions, a decrease in the Aldh7a1 expression level reduced carboxylic acid and betaine production, which indirectly intercepted pathways leading to oxidative stress and osmotic stress. In response to severe stress, compensatory mechanisms could be activated in the Rrm2b deleted kidney. One such mechanism is the proline generation and accumulation through the upregulation of P5cs and Pycr. Proline has also been reported to protect against oxidative stress, and its accumulation in cells is a response to physiological stress^36^.Another cellular compensation mechanism is enhanced Bhmt and reduced Dmgdh expression, both of which cause dimethylglycine accumulation and homocysteine consumption in the kidney, resulting in reduction of oxidative damage due to high levels of homocysteine. Previous reports have shown that homocysteine is a toxic compound to tissues in high concentrations^38^. Therefore, loss of Rrm2b in cells results in elevated oxidative stress and dysregulated metabolic activities, with subsequent activation of certain alternative compensatory pathways to counteract oxidative damage.

### The importance of Rrm2b in kidney

Mitochondria supply ATP, which is required for critical cellular functions throughout the body, especially in highly energy-dependent organs. Kidneys require a large amount of energy to filter minerals and other materials, perform tubular reabsorption and pass urine out of the body. Renal proximal tubules have abundant mitochondria to support ion transfer, such as that performed by the Na^+^-K^+^ ATPase pump. This study indicated that Rrm2b not only mediates mitochondrial DNA replication and repair but is also critical for the maintenance of mitochondrial functional integrity and metabolic activities. Using an Rrm2b-deficient (Rrm2b kiKO) mouse model, we propose that Rrm2b is essential for normal functions of the kidney.

## Supplementary information

Supplemental figures and Supplemental Tables

Supplemental Tables
