# Supplementary material for: Rrm2b deletion causes mitochondrial metabolic defects in renal tubules
Source: Sci Rep. 2019 Sep 13;9:13238. doi: 10.1038/s41598-019-49663-3 (PMC6744457; doi:10.1038/s41598-019-49663-3)
Supplement: Supplementary file 1 — Supplemental figures and Supplemental Tables [file 41598_2019_49663_MOESM1_ESM.pdf]

Supplementary information

Rrm2b deletion causes mitochondrial metabolic defects in renal tubules

Yi-Fan Chen[1], I-Hsuan Lin[2], Yu-Ru Guo[3], Wei-Jun Chiu[1], Mai-Szu Wu[4,5], Wei Jia[6], Yun Yen[2,3]

[1]The Ph.D. Program for Translational Medicine, College of Medical Science and Technology, Taipei Medical University, 11031 Taipei, Taiwan

[2]TMU Research Center of Cancer Translational Medicine, Taipei Medical University, 11031 Taipei, Taiwan

[3]The Ph.D. Program of Cancer Biology and Drug Discovery, College of Medical Science and Technology, Taipei Medical University, 11031 Taipei, Taiwan

[4]Department of Internal Medicine, School of Medicine, College of Medicine, Taipei Medical University, 11031 Taipei, Taiwan

[5]Division of Nephrology, Department of Internal Medicine, Taipei Medical University-Shuang Ho Hospital, 23561 New Taipei City, Taiwan

[6]Cancer Biology Program, University of Hawaii Cancer Center, Honolulu, HI 96813, US

Corresponding Author: Yun Yen, Taipei Medical University, 250 Wuxing Street, Taipei 11031, Taiwan. Phone: 8862-2736-1661, ext. 1588; Fax: 8862-2378-7795; E-mail: yyen@tmu.edu.tw

Supplemental Figure S1

a

Age	Genotype	BUN (mg/dL)	CREA (mg/dL)
3 mo	F/F	30.5 ± 10.7	0.33 ± 0.2
	KiKO	44.6 ± 13.3**	0.47 ± 0.2
6 mo	F/F	22.7 ± 3.9	0.32 ± 0.1
	KiKO	26.2 ± 6.4	0.28 ± 0.1

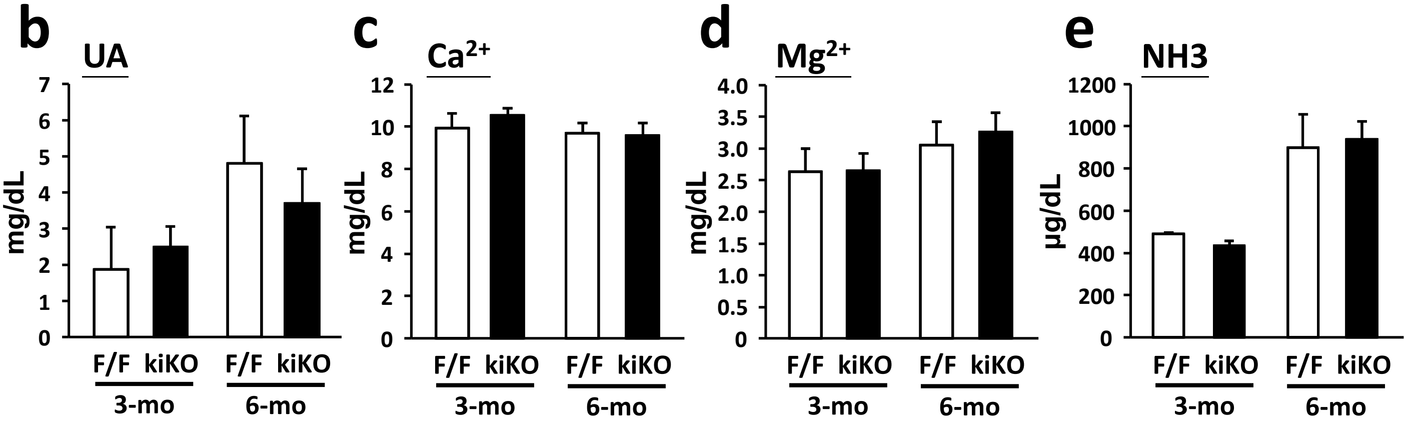

f

Genotype	Total protein (g/dL)	Creatinine (mg/dL)	Creatinine/ total protein
F/F	0.43 ± 0.19	78.0 ± 61.8	0.17 ± 0.06
KiKO	0.37 ± 0.06	49.3 ± 17.2	0.13 ± 0.03

Supplemental Figure S1_Continued

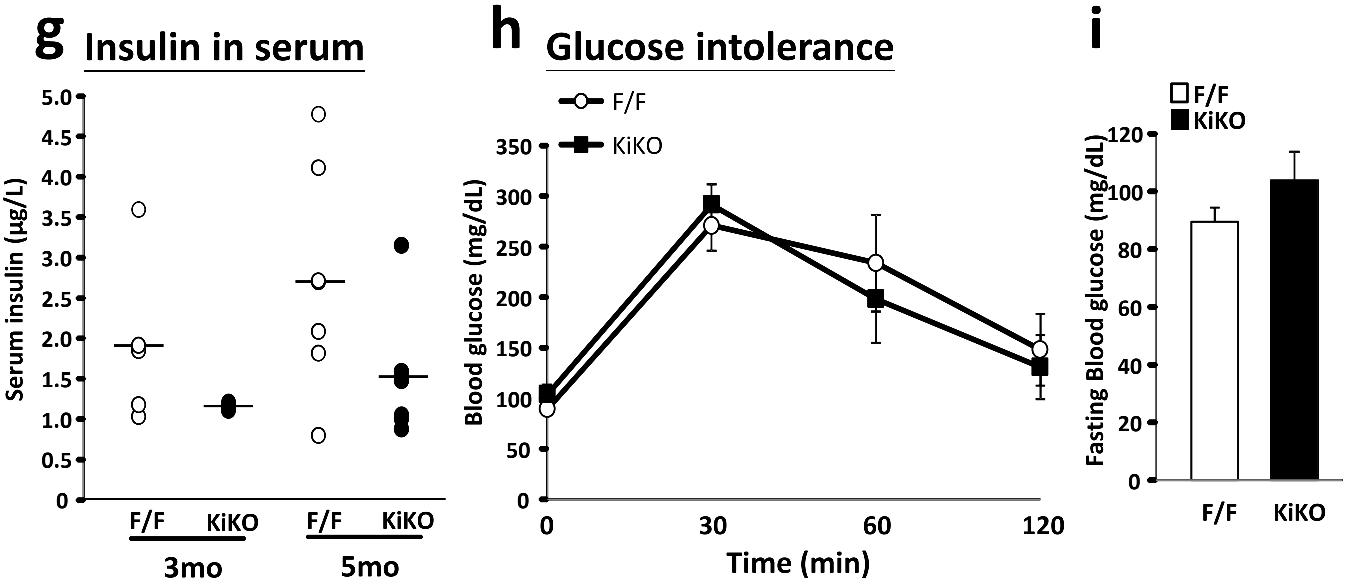

Supplemental Figure S1. Generation of the Rrm2b kidney-specific knockout mouse model and examination of biochemical indices. (a-e) The amount of blood urea nitrogen (BUN), creatinine (CREA), uric acid (UA), calcium (Ca^{2+}), magnesium (Mg^{2+}) and ammonia (NH_3) detected from mouse serum. **(f)** Mouse urines were collected and analyzed for total protein and creatinine using Fuji Dri-chem slide by Fuji Dri-chem 4000i (FUJIFIRM Corporation, Tokyo, Japan). 6 mice of each group were analyzed. **(g)** After two hours fasting, the insulin concentration was detected in serum from Rrm2b kiKO mice using Mouse insulin ELISA kit (Mercodia). **(h)** After 12 hours fasting, mice were treated with 1.5g glucose/kg body weight and monitored the blood glucose for 120 minutes. **(i)** Quantified data of blood glucose was determined after 12 hours fasting. The results are presented as the mean ± SD. *p<0.05; **p<0.005 ; ***p<0.001.

Supplemental Figure S2

a 3-mo male

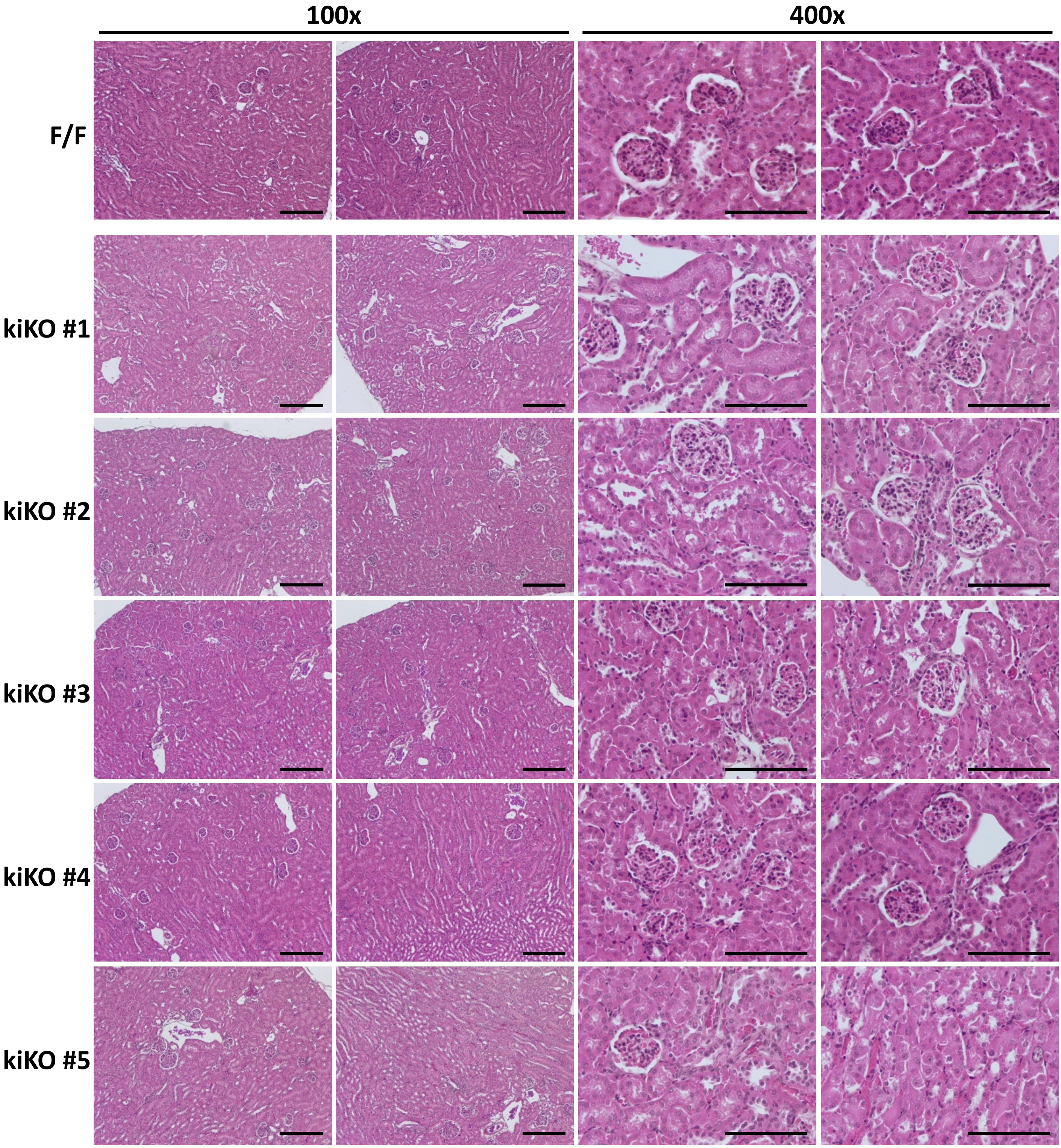

Supplemental Figure S2_Continued

b 5-mo male

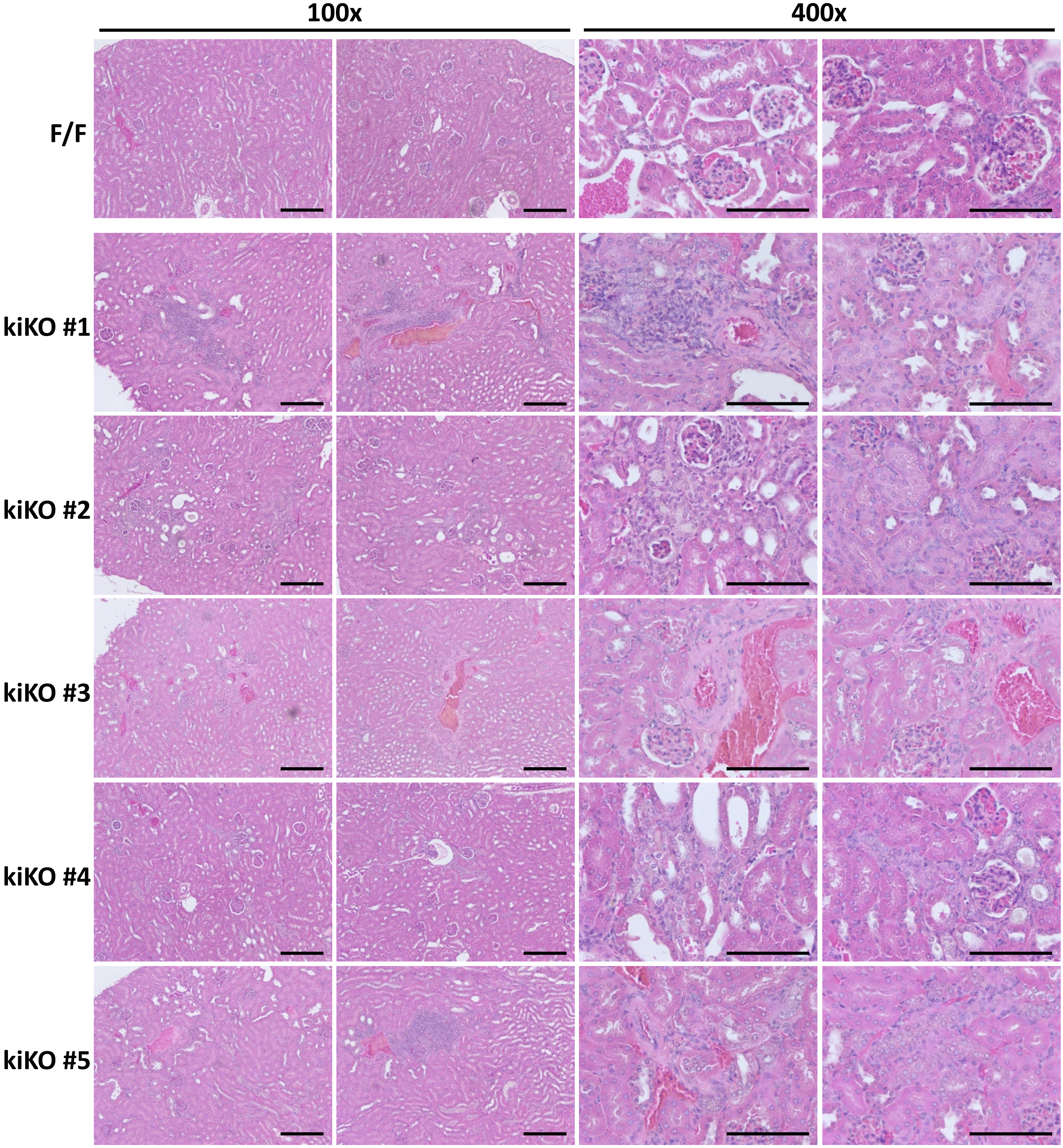

Supplemental Figure S2_Continued

c **5-mo female**

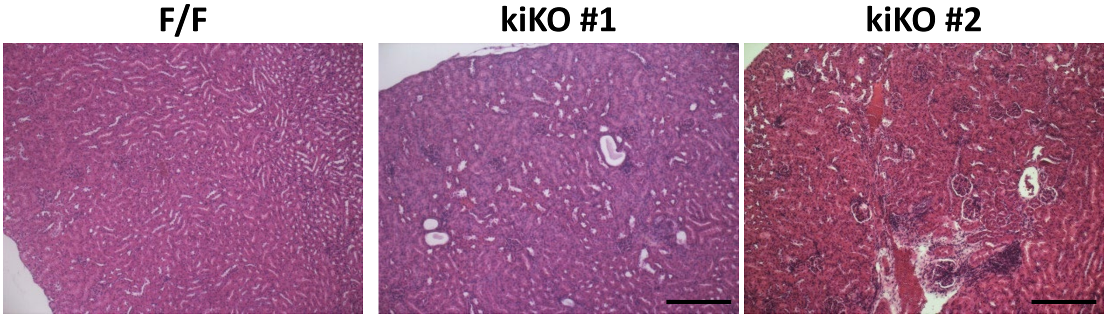

Supplemental Figure S2. Histopathological analysis for Rrm2b kiKO mice at 3 and 5 months of age. H&E staining of tissue sections from Rrm2b kiKO and control (F/F) **(a)** male mice at 3 months old. **(b)** male mice at 5 months old. **(c)** female mice at 5 months old. Scale bar, 100µm.

Supplemental Figure S3

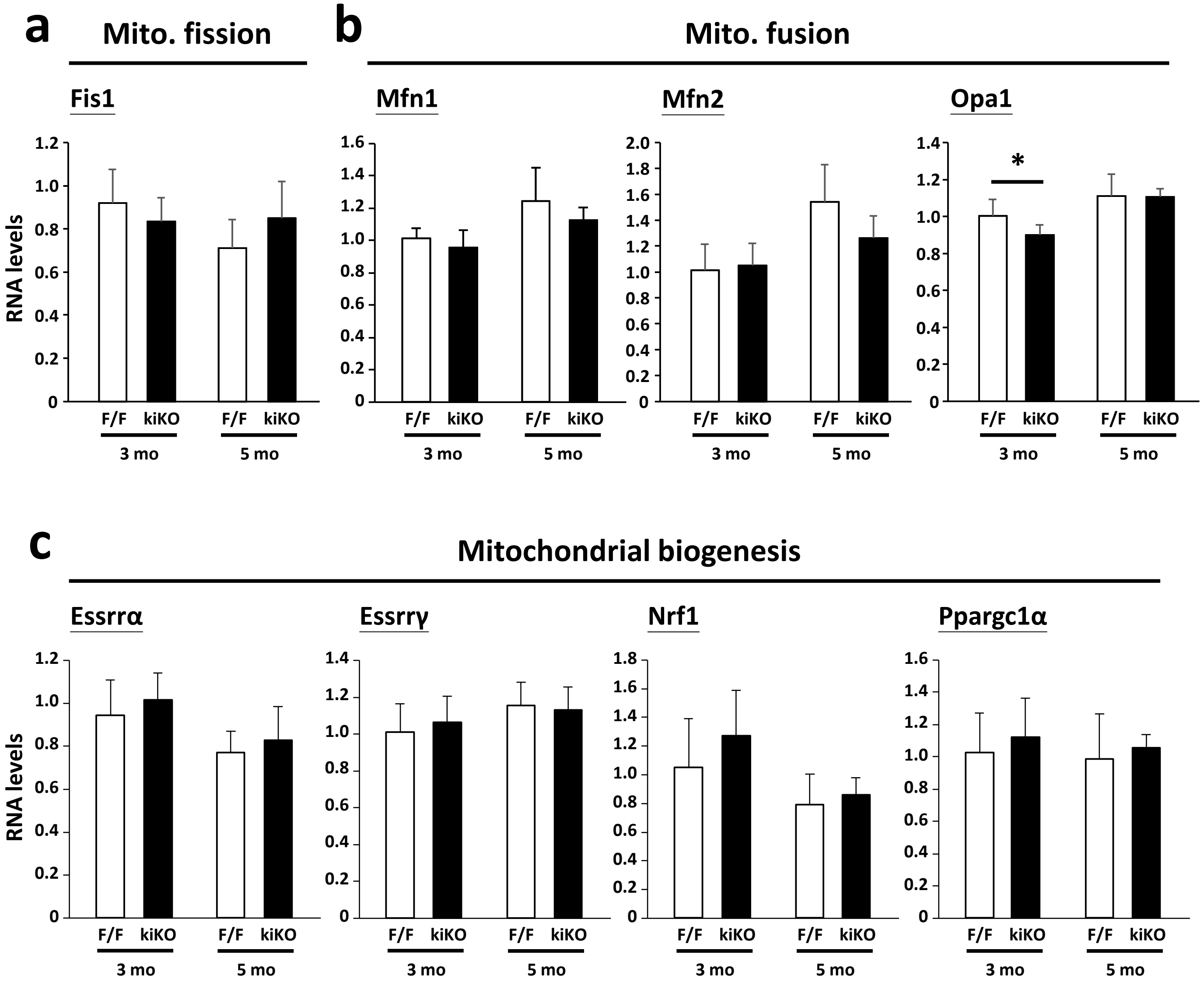

Supplemental Figure S3. Rrm2b deletion caused no apparent defects on mitochondrial fusion-fission and biogenesis. Gene expression levels of key genes related to **(a)** mitochondrial fusion, **(b)** mitochondrial fission, and **(c)** mitochondrial biogenesis. Fis1, fission, mitochondrial 1; Mfn1, mitofusin 1; Mfn2, mitofusin 2; Opa1, mitochondrial dynamin like GTPase; Essrrα, estrogen related receptor, alpha; Essrrγ, estrogen-related receptor gamma; Nrf1, nuclear respiratory factor 1; Ppargc1α, peroxisome proliferative activated receptor, gamma, coactivator 1 alpha.

Supplemental Figure S4

a

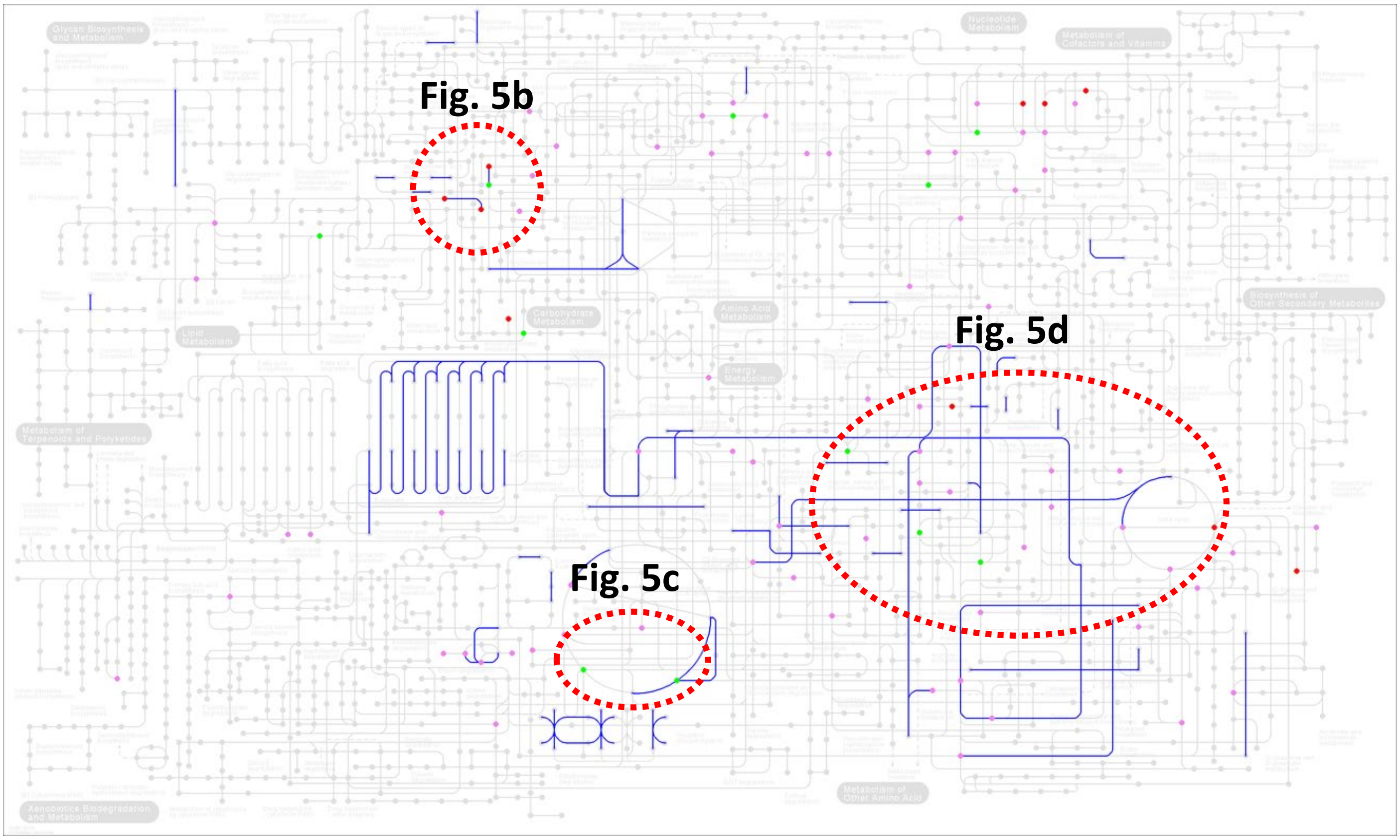

b

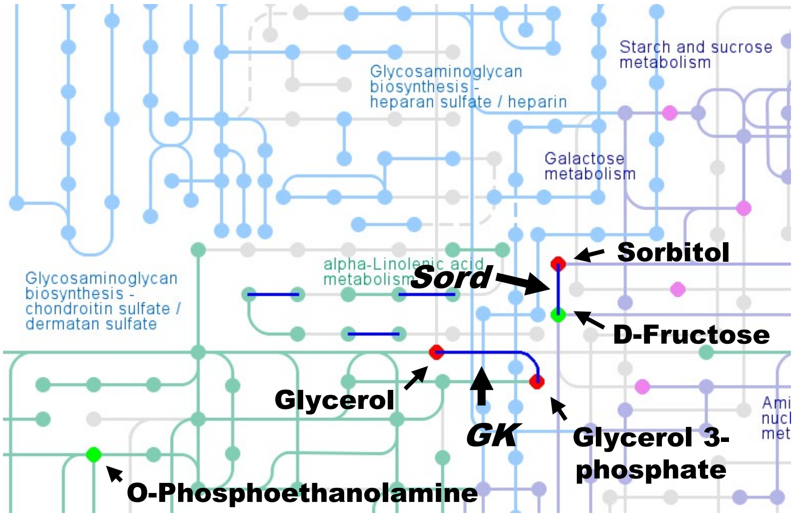

Supplemental Figure S4_Continued

c

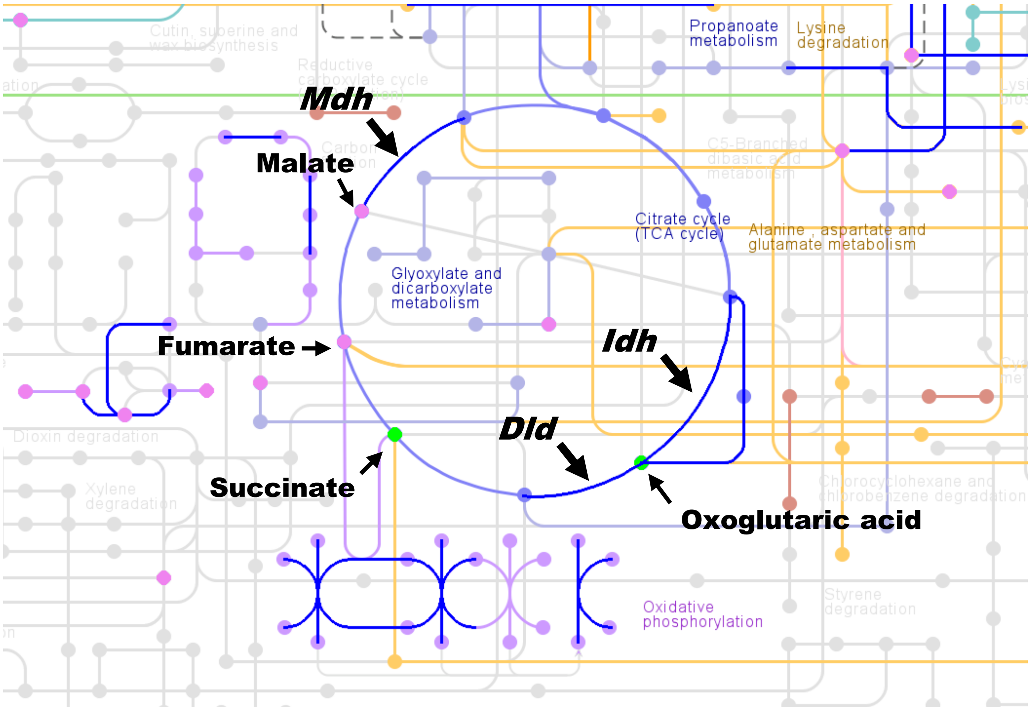

d

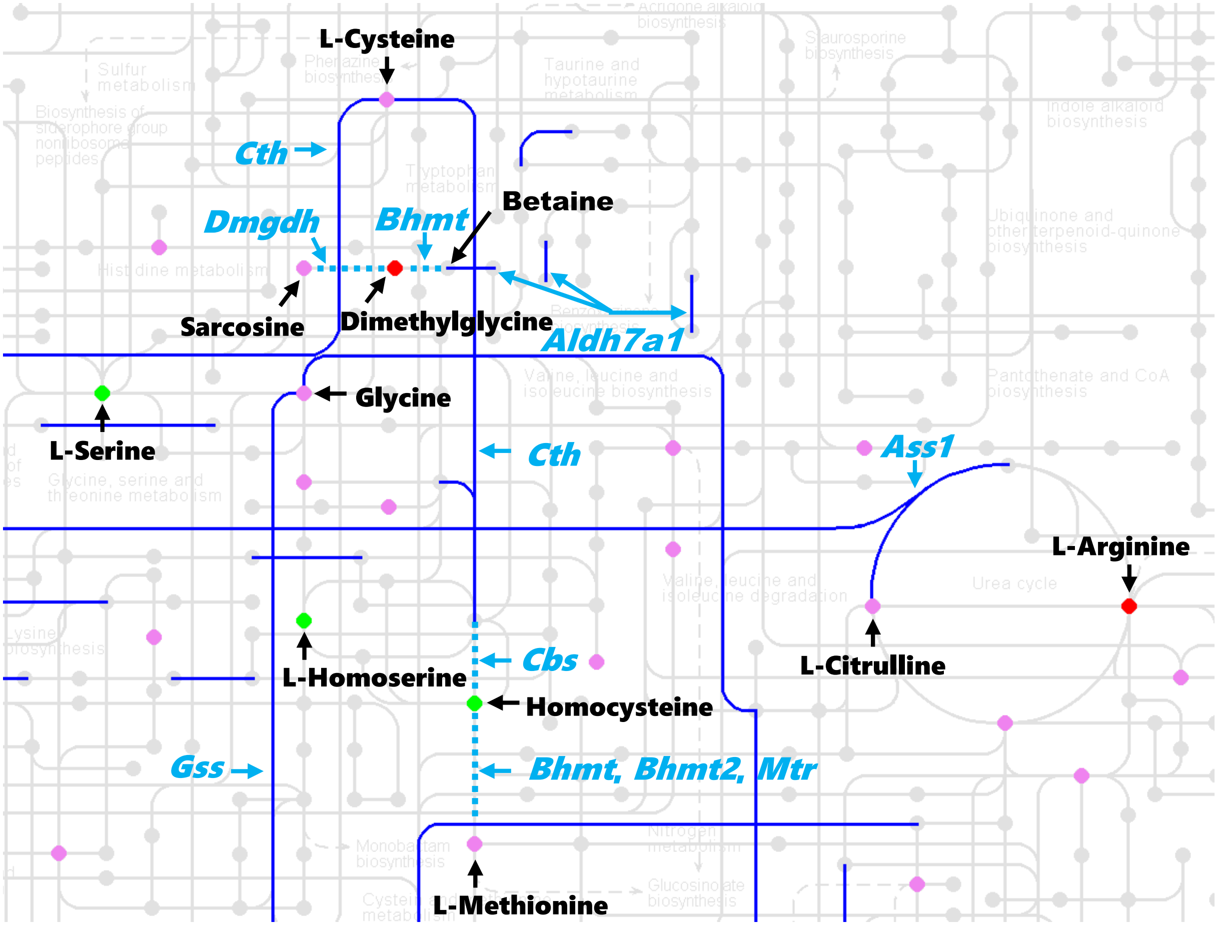

Supplemental Figure S4. Changes of KEGG mouse metabolic pathway in the Rrm2b kiKO mice. (a) KEGG mouse metabolic pathway overview (mmu01100) with candidate mitochondrial proteins and metabolites labeled. The enzymatic reactions carried out by mitochondrial proteins were highlighted in blue. Metabolites that showed higher and lower concentrations in kiKO were colored red and green respectively. Metabolites that showed no change in concentrations were colored purple. The zoomed-in view of three sections were shown in (b), (c) and (d). **(b)** The zoomed-in view of glycerol kinase and sorbitol dehydrogenase and their substrates and products on the KEGG mouse metabolic pathway overview (mmu01100). **(c)** The zoomed-in view of citrate cycle on the KEGG mouse metabolic pathway overview (mmu01100). **(d)** The zoomed-in view of methionine, homocysteine, betaine and glycine metabolism on the KEGG mouse metabolic pathway overview (mmu01100).

Supplemental Figure S5

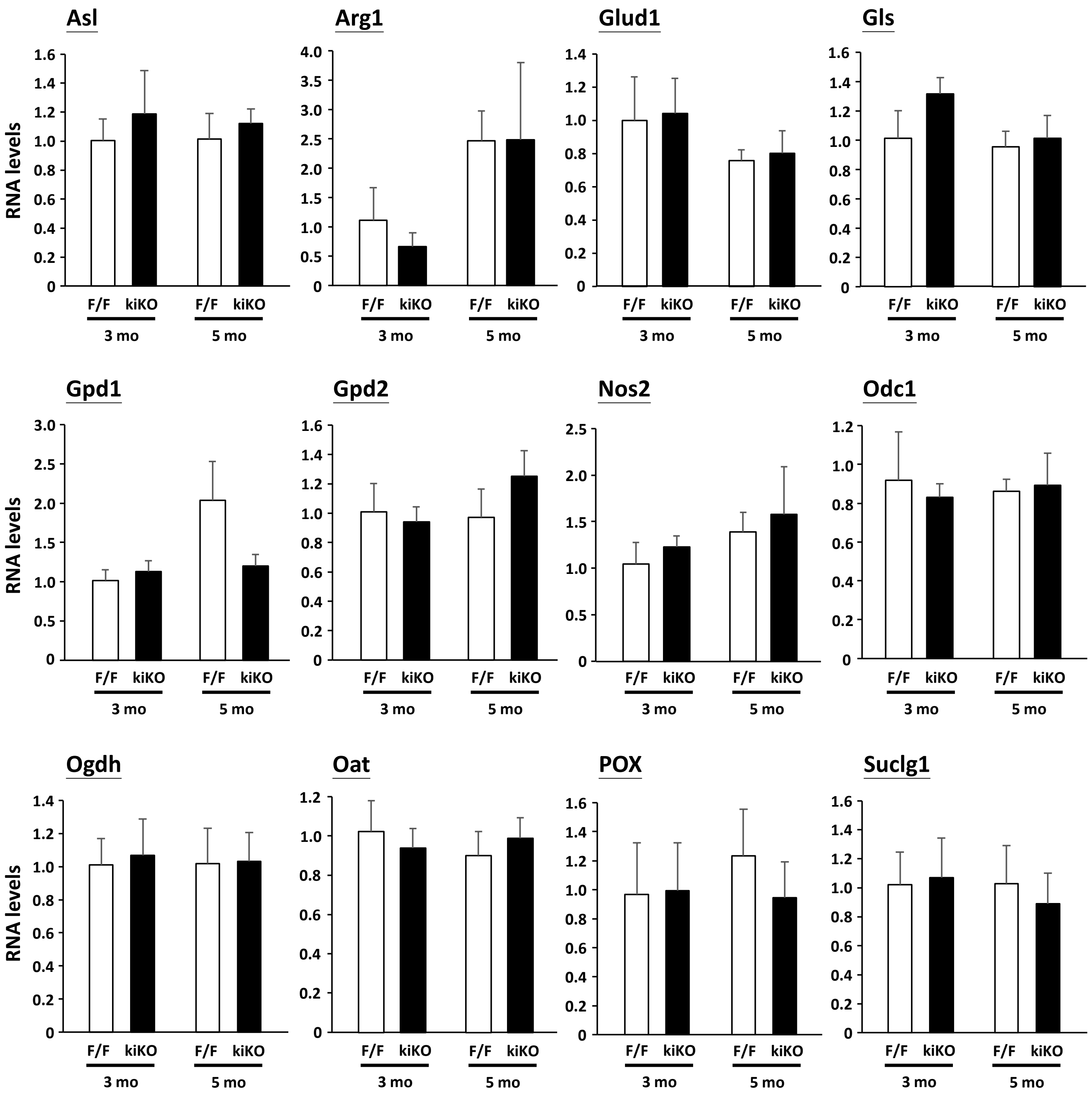

Supplemental Figure S5. No significant differences on the expression levels of the metabolic enzymes. The mRNA levels of the metabolic enzymes were determined by real-time quantitative PCR. Asl, argininosuccinate lyase; Arg1, Arginase; Glud1, Glutamate dehydrogenase; Gls, Glutaminase; Gpd1, glycerol-3-phosphate dehydrogenase 1 (soluble); Gpd2, glycerol phosphate dehydrogenase 2, mitochondrial; Nos2, Nitric oxide synthase; Odc1, Ornithine Decarboxylase; Ogdh, oxoglutarate (alpha-ketoglutarate) dehydrogenase (lipoamide), transcript variant 1; Oat, Ornithine aminotransferase; POX, Proline dehydrogenase/ Prolin oxidase; Suclg1, succinate-CoA ligase, GDP-forming, alpha subunit.
