# Supplementary material for: Rrm2b deletion causes mitochondrial metabolic defects in renal tubules
Source: Sci Rep. 2019 Sep 13;9:13238. doi: 10.1038/s41598-019-49663-3 (PMC6744457; doi:10.1038/s41598-019-49663-3)
Supplement: Supplementary file 2 — Supplemental Tables [file 41598_2019_49663_MOESM2_ESM.docx]

**Supplemental Table S1. Metabolomics**

| **Name** | **RT_Min** | **Cdh (Cdh16/+)** | **Rrm (Rrm2bF/F;Cdh16/+)** | **Cdh (log2 & Scaled)** | **Rrm (log2 & Scaled)** | **log2fc** | **HMDB** |
| --- | --- | --- | --- | --- | --- | --- | --- |
| L-Homoserine | 10.16 | 110.73 | 9.80 | -1.04 | -1.99 | -0.95 | HMDB0000719 |
| O-Phosphoethanolamine | 13.49 | 4552.65 | 848.44 | 0.60 | -0.09 | -0.70 | HMDB0000224 |
| Gluconolactone | 14.42 | 302.10 | 51.91 | -0.60 | -1.28 | -0.69 | HMDB0000150 |
| Pelargonic acid | 9.09 | 31.60 | 7.00 | -1.59 | -2.14 | -0.54 | HMDB0000847 |
| Adenine | 14.2 | 79.69 | 22.25 | -1.18 | -1.64 | -0.46 | HMDB0000034 |
| Oxoglutaric acid | 11.45 | 871.75 | 278.74 | -0.13 | -0.57 | -0.44 | HMDB0000208 |
| Cytidine | 20.21 | 953.91 | 347.67 | -0.09 | -0.47 | -0.39 | HMDB0000089 |
| L-Serine | 9.2 | 96764.21 | 43164.10 | 1.96 | 1.58 | -0.38 | HMDB0000187 |
| L-Sorbose | 14.42 | 8403.43 | 3448.85 | 0.88 | 0.50 | -0.37 | HMDB0001266 |
| Succinic acid | 8.61 | 22993.63 | 10058.61 | 1.32 | 0.96 | -0.36 | HMDB0000254 |
| Myoinositol | 16.16 | 16253.39 | 7695.30 | 1.17 | 0.84 | -0.32 | HMDB0000211 |
| D-Fructose | 14.47 | 2207.71 | 1110.92 | 0.28 | 0.02 | -0.26 | HMDB0000660 |
| Homocysteine | 12.33 | 39.35 | 17.15 | -1.50 | -1.75 | -0.26 | HMDB0000742 |
| Pyrophosphate | 7.86 | 437.89 | 232.18 | -0.43 | -0.65 | -0.21 | HMDB0000250 |
| Taurine | 12.46 | 30132.55 | 18985.12 | 1.44 | 1.23 | -0.21 | HMDB0000251 |
| Malic acid | 10.58 | 16713.14 | 10323.12 | 1.18 | 0.97 | -0.21 | HMDB0000744 |
| L-Aspartic acid | 10.93 | 107760.77 | 71652.29 | 2.00 | 1.79 | -0.21 | HMDB0000191 |
| Palmitic acid | 15.52 | 21362.62 | 13576.92 | 1.29 | 1.09 | -0.20 | HMDB0000220 |
| D-Ribose | 12.56 | 43447.76 | 29036.07 | 1.60 | 1.41 | -0.19 | HMDB0000283 |
| Niacinamide | 10.52 | 12092.21 | 7842.31 | 1.04 | 0.85 | -0.18 | HMDB0001406 |
| D-2-Hydroxyglutaric acid | 11.44 | 587.03 | 369.63 | -0.30 | -0.45 | -0.15 | HMDB0000606 |
| L-Glutamine | 13.35 | 217.97 | 133.65 | -0.74 | -0.88 | -0.14 | HMDB0000641 |
| Petroselinic acid | 16.82 | 3545.54 | 2428.64 | 0.49 | 0.35 | -0.14 | HMDB0002080 |
| Urea | 7.75 | 28977.43 | 21563.44 | 1.42 | 1.28 | -0.14 | HMDB0000294 |
| Adenosine | 19.81 | 955.86 | 622.80 | -0.09 | -0.23 | -0.14 | HMDB0000050 |
| D-Threitol | 10.84 | 337.60 | 211.64 | -0.55 | -0.68 | -0.14 | HMDB0004136 |
| L-Arabitol | 13 | 16073.89 | 11941.74 | 1.16 | 1.03 | -0.13 | HMDB0001851 |
| Decanoylcarnitine | 10.15 | 24.01 | 13.83 | -1.71 | -1.85 | -0.13 | HMDB0000651 |
| L-Phenylalanine | 12.01 | 6486.53 | 4699.23 | 0.76 | 0.63 | -0.13 | HMDB0000159 |
| L-Arabinose | 12.42 | 287.93 | 185.82 | -0.62 | -0.74 | -0.12 | HMDB0000646 |
| Methylcysteine | 9.87 | 295.19 | 191.03 | -0.61 | -0.73 | -0.12 | HMDB0002108 |
| Spermidine | 17.25 | 333.45 | 218.38 | -0.55 | -0.67 | -0.12 | HMDB0001257 |
| Guanosine | 20.66 | 1023.48 | 707.85 | -0.06 | -0.17 | -0.12 | HMDB0000133 |
| D-Glucose | 14.75 | 28555.41 | 22505.43 | 1.42 | 1.30 | -0.12 | HMDB0000122 |
| D-Galactose | 14.75 | 28555.41 | 22505.43 | 1.42 | 1.30 | -0.12 | HMDB0000143 |
| Citrulline | 13.78 | 468.73 | 316.38 | -0.40 | -0.51 | -0.11 | HMDB0000904 |
| L-Alanine | 6.09 | 70291.67 | 59731.57 | 1.81 | 1.72 | -0.10 | HMDB0000161 |
| D-Xylose | 12.35 | 218.24 | 149.14 | -0.74 | -0.83 | -0.09 | HMDB0000098 |
| 2-Hydroxypyridine | 5.33 | 240.50 | 168.48 | -0.70 | -0.78 | -0.09 | HMDB0013751 |
| Stearic acid | 17 | 11231.15 | 9439.99 | 1.00 | 0.93 | -0.07 | HMDB0000827 |
| Beta-Alanine | 9.91 | 771.24 | 604.97 | -0.18 | -0.24 | -0.06 | HMDB0000056 |
| L-Tyrosine | 14.81 | 20092.13 | 17931.77 | 1.26 | 1.20 | -0.06 | HMDB0000158 |
| Pyruvic acid | 5.47 | 3024.47 | 2526.43 | 0.42 | 0.37 | -0.05 | HMDB0000243 |
| Galactonic acid | 15.42 | 151.95 | 113.06 | -0.90 | -0.95 | -0.05 | HMDB0000565 |
| Cholesterol | 23.92 | 24925.19 | 22662.53 | 1.36 | 1.30 | -0.05 | HMDB0000067 |
| 2-Hydroxy-3-methylbutyric acid | 6.88 | 1730.43 | 1418.38 | 0.18 | 0.12 | -0.05 | HMDB0000407 |
| L-Proline | 8.48 | 31546.77 | 29041.33 | 1.46 | 1.41 | -0.05 | HMDB0000162 |
| L-Glutamic acid | 11.91 | 12972.47 | 11596.83 | 1.07 | 1.02 | -0.05 | HMDB0000148 |
| Dodecanoic acid | 12.11 | 102.43 | 75.83 | -1.07 | -1.12 | -0.05 | HMDB0000638 |
| MG(18:2(9Z,12Z)/0:0/0:0) | 20.42 | 57.11 | 41.52 | -1.33 | -1.38 | -0.05 | HMDB0011568 |
| Fumaric acid | 8.95 | 3037.44 | 2591.73 | 0.43 | 0.38 | -0.04 | HMDB0000134 |
| Sarcosine | 6.49 | 197.51 | 151.48 | -0.78 | -0.83 | -0.04 | HMDB0000271 |
| Palmitoleic acid | 15.36 | 235.16 | 187.33 | -0.71 | -0.74 | -0.03 | HMDB0003229 |
| L-Lysine | 14.66 | 12199.14 | 11403.08 | 1.04 | 1.01 | -0.03 | HMDB0000182 |
| Glycolic acid | 5.76 | 1732.61 | 1506.10 | 0.18 | 0.15 | -0.03 | HMDB0000115 |
| L-Valine | 7.48 | 27002.49 | 26264.63 | 1.39 | 1.37 | -0.03 | HMDB0000883 |
| L-Alpha-aminobutyric acid | 6.93 | 250.37 | 203.17 | -0.68 | -0.70 | -0.02 | HMDB0000452 |
| L-Methionine | 10.91 | 5665.30 | 5240.41 | 0.70 | 0.68 | -0.02 | HMDB0000696 |
| L-Leucine | 8.16 | 29302.88 | 28951.89 | 1.43 | 1.41 | -0.02 | HMDB0000687 |
| L-Alloisoleucine | 8.43 | 10605.08 | 10160.86 | 0.98 | 0.96 | -0.02 | HMDB0000557 |
| Heptadecanoic acid | 16.26 | 237.07 | 195.75 | -0.70 | -0.72 | -0.02 | HMDB0002259 |
| L-Threonine | 9.51 | 11707.36 | 11327.00 | 1.02 | 1.01 | -0.01 | HMDB0000167 |
| L-Pipecolic acid | 9.24 | 410.34 | 348.33 | -0.46 | -0.47 | -0.01 | HMDB0000716 |
| Caproic acid | 5.7 | 90.46 | 73.68 | -1.13 | -1.13 | -0.01 | HMDB0000535 |
| L-Cysteine | 11.28 | 8771.57 | 8566.38 | 0.89 | 0.89 | 0.00 | HMDB0000574 |
| Alpha-Tocopherol | 23.56 | 28.00 | 22.51 | -1.65 | -1.64 | 0.01 | HMDB0001893 |
| Aminoadipic acid | 12.79 | 158.74 | 137.08 | -0.88 | -0.87 | 0.01 | HMDB0000510 |
| L-Asparagine | 12.41 | 1727.63 | 1644.08 | 0.18 | 0.19 | 0.01 | HMDB0000168 |
| 2-Hydroxybutyric acid | 6.4 | 799.79 | 739.07 | -0.16 | -0.15 | 0.01 | HMDB0000008 |
| Ornithine | 13.76 | 4929.63 | 4897.83 | 0.64 | 0.65 | 0.01 | HMDB0000214 |
| Myristic acid | 13.88 | 188.06 | 165.24 | -0.80 | -0.79 | 0.01 | HMDB0000806 |
| Glycine | 8.6 | 52773.75 | 58290.27 | 1.69 | 1.71 | 0.02 | HMDB0000123 |
| Glutaric acid | 9.6 | 33.96 | 29.05 | -1.56 | -1.53 | 0.03 | HMDB0000661 |
| L-Histidine | 14.69 | 3436.68 | 3563.90 | 0.48 | 0.52 | 0.04 | HMDB0000177 |
| Linoleic acid | 16.79 | 1178.29 | 1179.44 | 0.01 | 0.05 | 0.04 | HMDB0000673 |
| Uric acid | 16.14 | 162.87 | 151.02 | -0.87 | -0.83 | 0.04 | HMDB0000289 |
| Behenic acid | 19.63 | 47.51 | 42.20 | -1.41 | -1.37 | 0.04 | HMDB0000944 |
| Pantothenic acid | 15.25 | 2439.49 | 2534.83 | 0.33 | 0.37 | 0.04 | HMDB0000210 |
| Pyrrole-2-carboxylic acid | 9.13 | 61.99 | 55.98 | -1.30 | -1.25 | 0.04 | HMDB0004230 |
| Alpha-Lactose | 20.39 | 33.96 | 30.01 | -1.56 | -1.52 | 0.05 | HMDB0000186 |
| Gamma-Aminobutyric acid | 11.02 | 5535.03 | 6002.82 | 0.69 | 0.74 | 0.05 | HMDB0000112 |
| Benzoic acid | 7.84 | 660.65 | 668.58 | -0.25 | -0.20 | 0.05 | HMDB0001870 |
| 3-hydroxypyridine | 6.7 | 134.27 | 127.72 | -0.95 | -0.90 | 0.05 |  |
| Putrescine | 13.16 | 227.82 | 223.89 | -0.72 | -0.66 | 0.06 | HMDB0001414 |
| Hypoxanthine | 13.68 | 10361.29 | 11905.69 | 0.97 | 1.03 | 0.06 | HMDB0000157 |
| Elaidic acid | 16.87 | 417.56 | 432.07 | -0.45 | -0.38 | 0.07 | HMDB0000573 |
| Oleic acid | 16.86 | 417.56 | 432.07 | -0.45 | -0.38 | 0.07 | HMDB0000207 |
| D-Mannose | 14.61 | 10632.94 | 12717.59 | 0.98 | 1.06 | 0.08 | HMDB0000169 |
| Tetracosanoic acid | 20.83 | 84.19 | 83.51 | -1.16 | -1.08 | 0.08 | HMDB0002003 |
| 5-Hydroxydopamine | 16.9 | 70.95 | 71.84 | -1.24 | -1.14 | 0.09 | HMDB0004817 |
| Sucrose | 19.99 | 61.16 | 62.17 | -1.30 | -1.21 | 0.10 | HMDB0000258 |
| Uracil | 8.93 | 4959.09 | 5997.94 | 0.64 | 0.74 | 0.10 | HMDB0000300 |
| Rhamnose | 13.04 | 166.84 | 179.04 | -0.86 | -0.76 | 0.10 | HMDB0000849 |
| Glyceric acid | 8.85 | 65.38 | 68.13 | -1.27 | -1.17 | 0.10 | HMDB0000139 |
| Alpha-ketoisovaleric acid | 6.16 | 239.78 | 264.63 | -0.70 | -0.59 | 0.11 | HMDB0000019 |
| 1,5-Anhydrosorbitol | 14.1 | 437.79 | 495.84 | -0.43 | -0.32 | 0.11 | HMDB0002712 |
| Arachidic acid | 18.36 | 81.44 | 87.09 | -1.17 | -1.06 | 0.11 | HMDB0002212 |
| 3-Aminoisobutanoic acid | 10.3 | 46.18 | 53.62 | -1.43 | -1.27 | 0.16 | HMDB0003911 |
| Arachidonic acid | 17.93 | 664.25 | 874.07 | -0.25 | -0.08 | 0.17 | HMDB0001043 |
| D-Glucuronic acid | 14.91 | 12941.28 | 20873.72 | 1.07 | 1.27 | 0.20 | HMDB0000127 |
| Uridine | 18.61 | 1597.14 | 2461.32 | 0.14 | 0.36 | 0.22 | HMDB0000296 |
| L-Lactic acid | 5.61 | 33293.01 | 58846.58 | 1.48 | 1.71 | 0.23 | HMDB0000190 |
| Docosahexaenoic acid | 19.2 | 1061.88 | 1648.30 | -0.04 | 0.19 | 0.23 | HMDB0002183 |
| Xanthine | 15.49 | 1147.76 | 1787.88 | 0.00 | 0.22 | 0.23 | HMDB0000292 |
| Gluconic acid | 15.44 | 44.53 | 61.72 | -1.44 | -1.21 | 0.23 | HMDB0000625 |
| Creatinine | 11.33 | 1854.67 | 3210.24 | 0.21 | 0.47 | 0.26 | HMDB0000562 |
| Xanthosine | 20.1 | 162.69 | 262.18 | -0.87 | -0.59 | 0.28 | HMDB0000299 |
| L-Cystine | 17.54 | 4339.60 | 7956.99 | 0.58 | 0.86 | 0.28 | HMDB0000192 |
| Ribonolactone | 12.56 | 826.07 | 1546.01 | -0.15 | 0.16 | 0.31 | HMDB0001900 |
| Sorbitol | 14.91 | 9457.11 | 19595.62 | 0.93 | 1.24 | 0.31 | HMDB0000247 |
| Glycerol 3-phosphate | 13.3 | 3851.05 | 8350.10 | 0.53 | 0.88 | 0.35 | HMDB0000126 |
| Dimethylglycine | 4.79 | 4627.49 | 10150.40 | 0.61 | 0.96 | 0.35 | HMDB0000092 |
| Mannitol | 14.85 | 302.53 | 662.13 | -0.59 | -0.20 | 0.39 | HMDB0000765 |
| L-Arginine | 11.84 | 128.37 | 294.49 | -0.97 | -0.54 | 0.43 | HMDB0000517 |
| Inosine | 19.43 | 3034.79 | 9157.51 | 0.43 | 0.92 | 0.49 | HMDB0000195 |
| 7-Methylxanthine | 15.73 | 89.31 | 460.75 | -1.13 | -0.35 | 0.78 | HMDB0001991 |
| Pyroglutamic acid | 10.98 | 4707.01 | 30107.15 | 0.62 | 1.42 | 0.81 | HMDB0000267 |
| Glycerol | 8.19 | 2039.21 | 16019.62 | 0.25 | 1.16 | 0.91 | HMDB0000131 |
| Glucose 6-phosphate | 17.88 | 15.40 | 320.85 | -1.91 | -0.51 | 1.40 | HMDB0001401 |

**Supplemental Table S2. Mitochondrial Proteins**

| **Ensembl Primary Identifier** | **GenomeDB Identifier** | **Gene Symbol** | **NCBI Gene ID** | **Description** |
| --- | --- | --- | --- | --- |
| ENSMUSG00000057228 | MGI:1345167 | Aadat | 23923 | aminoadipate aminotransferase |
| ENSMUSG00000068522 | MGI:2181621 | Aard | 239435 | alanine and arginine rich domain containing protein |
| ENSMUSG00000010651 | MGI:3605455 | Acaa1b | 235674 | acetyl-Coenzyme A acyltransferase 1B |
| ENSMUSG00000062908 | MGI:87867 | Acadm | 11364 | acyl-Coenzyme A dehydrogenase, medium chain |
| ENSMUSG00000033533 | MGI:2152200 | Acsm1 | 117147 | acyl-CoA synthetase medium-chain family member 1 |
| ENSMUSG00000031785 | MGI:1340051 | Adgrg1 | 14766 | adhesion G protein-coupled receptor G1 |
| ENSMUSG00000074207 | MGI:87921 | Adh1 | 11522 | alcohol dehydrogenase 1 (class I) |
| ENSMUSG00000030088 | MGI:1340024 | Aldh1l1 | 107747 | aldehyde dehydrogenase 1 family, member L1 |
| ENSMUSG00000028737 | MGI:2443883 | Aldh4a1 | 212647 | aldehyde dehydrogenase 4 family, member A1 |
| ENSMUSG00000053644 | MGI:108186 | Aldh7a1 | 110695 | aldehyde dehydrogenase family 7, member A1 |
| ENSMUSG00000026687 | MGI:1861622 | Aldh9a1 | 56752 | aldehyde dehydrogenase 9, subfamily A1 |
| ENSMUSG00000028307 | MGI:87995 | Aldob | 230163 | aldolase B, fructose-bisphosphate |
| ENSMUSG00000039062 | MGI:5000466 | Anpep | 16790 | alanyl (membrane) aminopeptidase |
| ENSMUSG00000021866 | MGI:108481 | Anxa11 | 11744 | annexin A11 |
| ENSMUSG00000076441 | MGI:88090 | Ass1 | 11898 | argininosuccinate synthetase 1 |
| ENSMUSG00000052459 | MGI:1201780 | Atp6v1a | 11964 | ATPase, H+ transporting, lysosomal V1 subunit A |
| ENSMUSG00000019210 | MGI:894326 | Atp6v1e1 | 11973 | ATPase, H+ transporting, lysosomal V1 subunit E1 |
| ENSMUSG00000037685 | MGI:1330848 | Atp8a1 | 11980 | ATPase, aminophospholipid transporter (APLT), class I, type 8A, member 1 |
| ENSMUSG00000056124 | MGI:1928380 | B4galt6 | 56386 | UDP-Gal:betaGlcNAc beta 1,4-galactosyltransferase, polypeptide 6 |
| ENSMUSG00000018446 | MGI:1194505 | C1qbp | 12261 | complement component 1, q subcomponent binding protein |
| ENSMUSG00000001119 | MGI:88459 | Col6a1 | 12833 | collagen, type VI, alpha 1 |
| ENSMUSG00000028179 | MGI:1339968 | Cth | 107869 | cystathionase (cystathionine gamma-lyase) |
| ENSMUSG00000006932 | MGI:88276 | Ctnnb1 | 12387 | catenin (cadherin associated protein), beta 1 |
| ENSMUSG00000063694 | MGI:88578 | Cycs | 13063 | cytochrome c, somatic |
| ENSMUSG00000019929 | MGI:94872 | Dcn | 13179 | decorin |
| ENSMUSG00000020664 | MGI:107450 | Dld | 13382 | dihydrolipoamide dehydrogenase |
| ENSMUSG00000004789 | MGI:1926170 | Dlst | 78920 | dihydrolipoamide S-succinyltransferase (E2 component of 2-oxo-glutarate complex) |
| ENSMUSG00000021417 | MGI:1346064 | Eci2 | 23986 | enoyl-Coenzyme A delta isomerase 2 |
| ENSMUSG00000029163 | MGI:1926189 | Emilin1 | 100952 | elastin microfibril interfacer 1 |
| ENSMUSG00000052397 | MGI:98931 | Ezr | 22350 | ezrin |
| ENSMUSG00000069805 | MGI:95492 | Fbp1 | 14121 | fructose bisphosphatase 1 |
| ENSMUSG00000059363 | MGI:1096879 | Fxn | 14297 | frataxin |
| ENSMUSG00000038843 | MGI:95676 | Gcnt1 | 14537 | glucosaminyl (N-acetyl) transferase 1, core 2 |
| ENSMUSG00000025059 | MGI:106594 | Gk | 14933 | glycerol kinase |
| ENSMUSG00000026473 | MGI:95739 | Glul | 14645 | glutamate-ammonia ligase (glutamine synthetase) |
| ENSMUSG00000000594 | MGI:95762 | Gm2a | 14667 | GM2 ganglioside activator protein |
| ENSMUSG00000023019 | MGI:95679 | Gpd1 | 14555 | glycerol-3-phosphate dehydrogenase 1 (soluble) |
| ENSMUSG00000051043 | MGI:1917605 | Gprc5c | 70355 | G protein-coupled receptor, family C, group 5, member C |
| ENSMUSG00000025950 | MGI:96413 | Idh1 | 15926 | isocitrate dehydrogenase 1 (NADP+), soluble |
| ENSMUSG00000041248 | MGI:1927248 | Kcnj1 | 56379 | potassium inwardly-rectifying channel, subfamily J, member 1 |
| ENSMUSG00000023043 | MGI:96692 | Krt18 | 16668 | keratin 18 |
| ENSMUSG00000023039 | MGI:96704 | Krt7 | 110310 | keratin 7 |
| ENSMUSG00000019179 | MGI:97050 | Mdh2 | 17448 | malate dehydrogenase 2, NAD (mitochondrial) |
| ENSMUSG00000030621 | MGI:1916679 | Me3 | 109264 | malic enzyme 3, NADP(+)-dependent, mitochondrial |
| ENSMUSG00000054641 | MGI:1918195 | Mmrn1 | 70945 | multimerin 1 |
| ENSMUSG00000067818 | MGI:2138915 | Myl9 | 98932 | myosin, light polypeptide 9, regulatory |
| ENSMUSG00000029632 | MGI:107686 | Ndufa4 | 17992 | NADH dehydrogenase (ubiquinone) 1 alpha subcomplex, 4 |
| ENSMUSG00000034875 | MGI:94203 | Nudt19 | 110959 | nudix (nucleoside diphosphate linked moiety X)-type motif 19 |
| ENSMUSG00000063931 | MGI:97542 | Pepd | 18624 | peptidase D |
| ENSMUSG00000028691 | MGI:99523 | Prdx1 | 18477 | peroxiredoxin 1 |
| ENSMUSG00000036892 | MGI:1929093 | Prodh2 | 56189 | proline dehydrogenase (oxidase) 2 |
| ENSMUSG00000104445 | MGI:1927379 | Rhbg | 58176 | Rhesus blood group-associated B glycoprotein |
| ENSMUSG00000061904 | MGI:1353498 | Slc25a3 | 18674 | solute carrier family 25 (mitochondrial carrier, phosphate carrier), member 3 |
| ENSMUSG00000022003 | MGI:1914804 | Slc25a30 | 67554 | solute carrier family 25, member 30 |
| ENSMUSG00000050144 | MGI:2444391 | Slc25a44 | 229517 | solute carrier family 25, member 44 |
| ENSMUSG00000016319 | MGI:1353496 | Slc25a5 | 11740 | solute carrier family 25 (mitochondrial carrier, adenine nucleotide translocator), member 5 |
| ENSMUSG00000027359 | MGI:1347099 | Slc27a2 | 26458 | solute carrier family 27 (fatty acid transporter), member 2 |
| ENSMUSG00000024131 | MGI:1195264 | Slc3a1 | 20532 | solute carrier family 3, member 1 |
| ENSMUSG00000060681 | MGI:2443511 | Slc9a6 | 236794 | solute carrier family 9 (sodium/hydrogen exchanger), member 6 |
| ENSMUSG00000027227 | MGI:98266 | Sord | 20322 | sorbitol dehydrogenase |
| ENSMUSG00000001750 | MGI:1350931 | Tcirg1 | 27060 | T cell, immune regulator 1, ATPase, H+ transporting, lysosomal V0 protein A3 |
| ENSMUSG00000020432 | MGI:98534 | Tcn2 | 21452 | transcobalamin 2 |
| ENSMUSG00000023456 | MGI:98797 | Tpi1 | 21991 | triosephosphate isomerase 1 |
| ENSMUSG00000025651 | MGI:107876 | Uqcrc1 | 22273 | ubiquinol-cytochrome c reductase core protein 1 |
| ENSMUSG00000063882 | MGI:1913826 | Uqcrh | 66576 | ubiquinol-cytochrome c reductase hinge protein |
| ENSMUSG00000023951 | MGI:103178 | Vegfa | 22339 | vascular endothelial growth factor A |
| ENSMUSG00000026728 | MGI:98932 | Vim | 22352 | vimentin |

**Supplemental Table S3. KEGG Pathway Mapping**

| **KEGG Pathway ID** | **KEGG Pathway Name** | **Number of Objects** | **Differential Abundant Compounds** | **Genes** |
| --- | --- | --- | --- | --- |
| mmu01100 | Metabolic pathways | 53 | 7-Methylxanthine, Adenine, Creatinine, Cytidine, D-Fructose, Dimethylglycine, Gluconolactone, Glucose 6-phosphate, Glycerol, Glycerol 3-phosphate, Homocysteine, Inosine, L-Arginine, L-Homoserine, L-Serine, Myoinositol, O-Phosphoethanolamine, Oxoglutaric acid, Sorbitol, Succinic acid, Xanthosine | Aadat, Acaa1b, Acadm, Acsm1, Adh1, Aldh4a1, Aldh7a1, Aldh9a1, Aldob, Anpep, Ass1, Atp6v1a, Atp6v1e1, B4galt6, Cth, Cycs, Dld, Dlst, Fbp1, Gcnt1, Gk, Glul, Idh1, Mdh2, Me3, Ndufa4, Prodh2, Sord, Tcirg1, Tpi1, Uqcrc1, Uqcrh |
| mmu01200 | Carbon metabolism | 13 | Gluconolactone, L-Serine, Oxoglutaric acid, Succinic acid | Acadm; Aldob; Dld; Dlst; Fbp1; Idh1; Mdh2; Me3; Tpi1 |
| mmu01230 | Biosynthesis of amino acids | 11 | Homocysteine, L-Arginine, L-Serine, O-Phosphoethanolamine, Oxoglutaric acid | Aldob; Ass1; Cth; Glul; Idh1; Tpi1 |
| mmu02010 | ABC transporters | 9 | D-Fructose, Glycerol, Glycerol 3-phosphate, L-Arginine, L-Cystine, L-Serine, Mannitol, Myoinositol, Sorbitol |  |
| mmu00010 | Glycolysis / Gluconeogenesis | 7 |  | Adh1; Aldh7a1; Aldh9a1; Aldob; Dld; Fbp1; Tpi1 |
| mmu00190 | Oxidative phosphorylation | 7 | Succinate | Atp6v1a; Atp6v1e1; Ndufa4; Tcirg1; Uqcrc1; Uqcrh |
| mmu00051 | Fructose and mannose metabolism | 7 | D-Fructose, Mannitol, Sorbitol | Aldob; Fbp1; Sord; Tpi1 |
| mmu00020 | Citrate cycle (TCA cycle) | 6 | Oxoglutaric acid, Succinic acid | Dld; Dlst; Idh1; Mdh2 |
| mmu00330 | Arginine and proline metabolism | 6 | Creatinine, L-Arginine | Aldh4a1; Aldh7a1; Aldh9a1; Prodh2 |
| mmu00620 | Pyruvate metabolism | 6 | Succinate | Aldh7a1; Aldh9a1; Dld; Mdh2; Me3 |
| mmu00630 | Glyoxylate and dicarboxylate metabolism | 6 | L-Serine, Oxoglutaric acid, Succinic acid | Dld; Glul; Mdh2 |
| mmu00270 | Cysteine and methionine metabolism | 6 | Homocysteine, L-Cystine, L-Homoserine, L-Serine | Cth, Mdh2 |
| mmu00071 | Fatty acid degradation | 6 |  | Acaa1b; Acadm; Adh1; Aldh7a1; Aldh9a1; Eci2 |
| mmu00260 | Glycine, serine and threonine metabolism | 6 | Dimethylglycine, L-Homoserine, L-Serine | Aldh7a1; Cth; Dld |
| mmu04146 | Peroxisome | 6 |  | Acaa1b; Eci2; Idh1; Nudt19; Prdx1; Slc27a2 |
